# Supplementary figures and images for: The wtf meiotic driver gene family has unexpectedly persisted for over 100 million years
Source: eLife. 2022 Oct 13;11:e81149. doi: 10.7554/eLife.81149 (PMC9562144; doi:10.7554/eLife.81149)

## GARD analysis of *S. octosporus* wtf genes

\*\*\*

seg355-1008

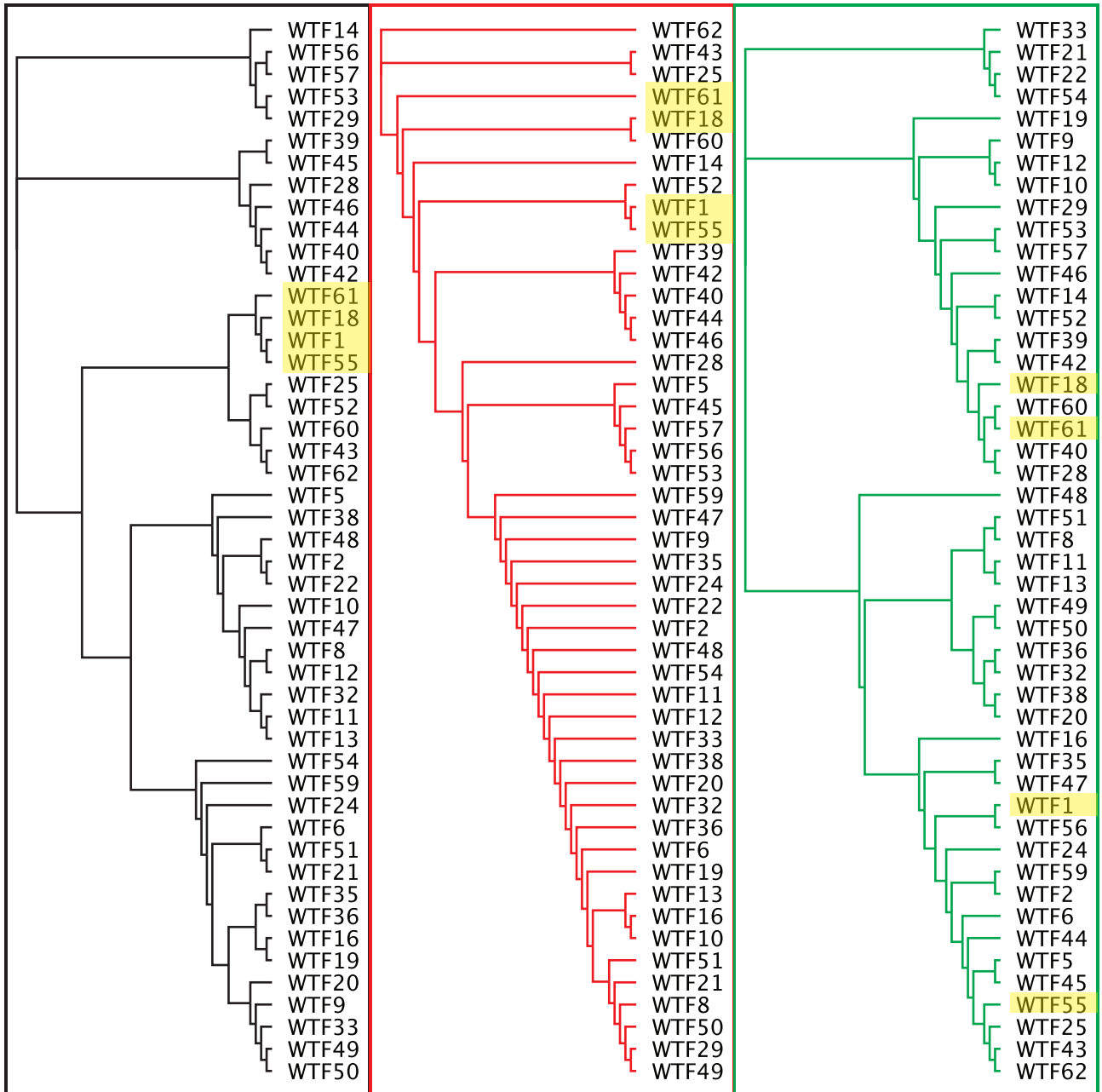

\*\*\* p-value= 0.0004

Supplement: Figure 5—figure supplement 2—source data 1. — GARD analysis of S. octosporus wtf predicted meiotic drivers and suppressors. This analysis found that a hypothesis allowing multiple trees for different segments of the alignment is >100 times more likely than a hypothesis allowing only a single tree, supporting that non-allelic recombination has occurred within the gene family. The analysis identified two likely breakpoints corresponding to positions 204 and 355 in the alignment, yielding three segments as depicted by the colored rectangles at the top of the figure. Both breakpoints have strong statistical support (***; p<0.0004). The trees generated for each segment (below) are distinct. The yellow highlighting is to help illustrate the incongruence between the trees. [file elife-81149-fig5-figsupp2-data1.pdf]

GARD analysis of *S. osmophilus* wtf genes

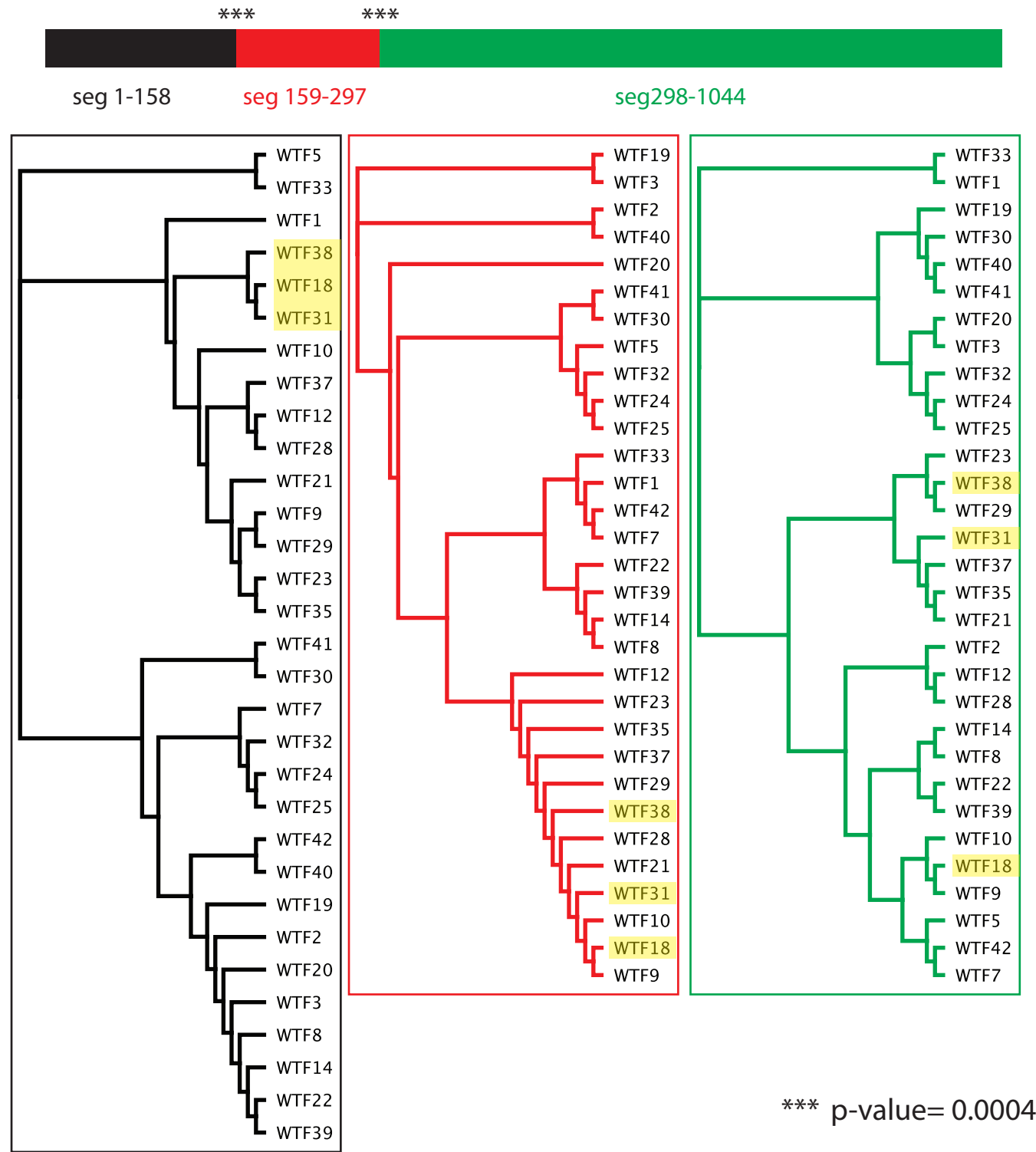

Supplement: Figure 5—figure supplement 2—source data 2. — GARD analysis of S. osmophilus wtf predicted meiotic drivers and suppressors. This analysis found that a hypothesis allowing multiple trees for different segments of the alignment is >100 times more likely than a hypothesis allowing only a single tree, supporting that non-allelic recombination has occurred within the gene family. The analysis identified two likely breakpoints corresponding to positions 159 and 298 in the alignment, yielding three segments as depicted by the colored rectangles at the top of the figure. Both breakpoints have strong statistical support (***; p<0.0004). The trees generated for each segment (below) are distinct. The yellow highlighting is to help illustrate the incongruence between the trees. [file elife-81149-fig5-figsupp2-data2.pdf]
