# Supplementary material for: The wtf meiotic driver gene family has unexpectedly persisted for over 100 million years
Source: eLife. 2022 Oct 13;11:e81149. doi: 10.7554/eLife.81149 (PMC9562144; doi:10.7554/eLife.81149)

Wild type cross

YEST plate

YEST plate

DY44286 × DY44287
2019-1
Successful octad: 10

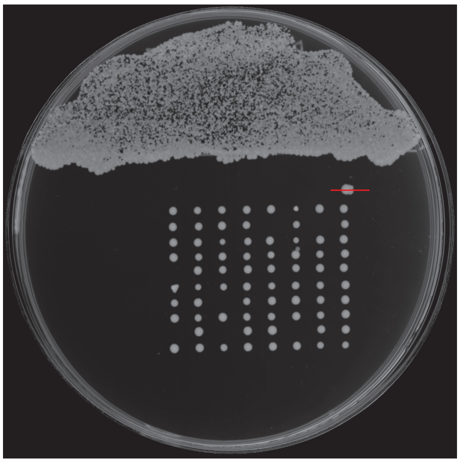

DY44286 × DY44287
2019-5
Successful octad: 11

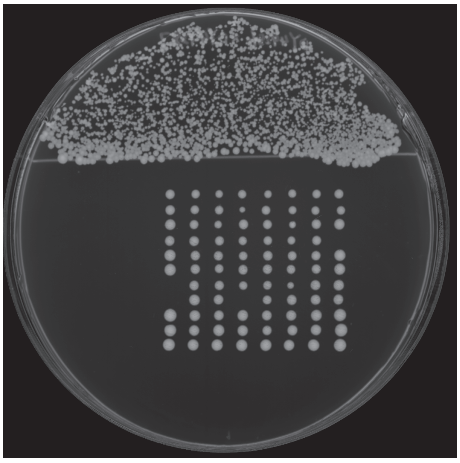

DY44286 × DY44287
2019-2
Successful octad: 11

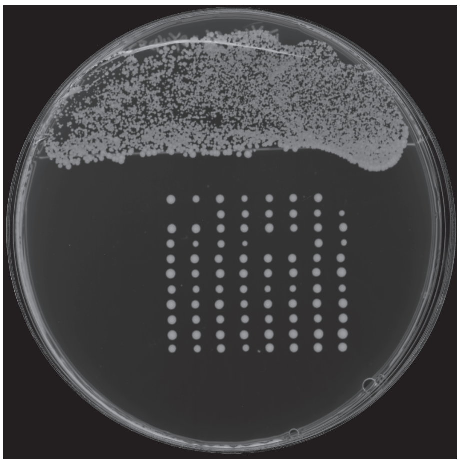

DY44286 × DY44287
2020-1
Successful octad: 11

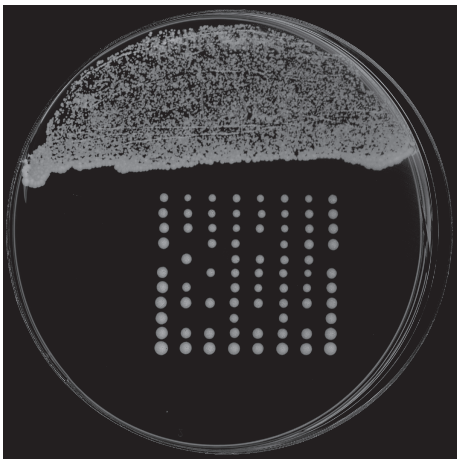

DY44286 × DY44287
2019-3
Successful octad: 11

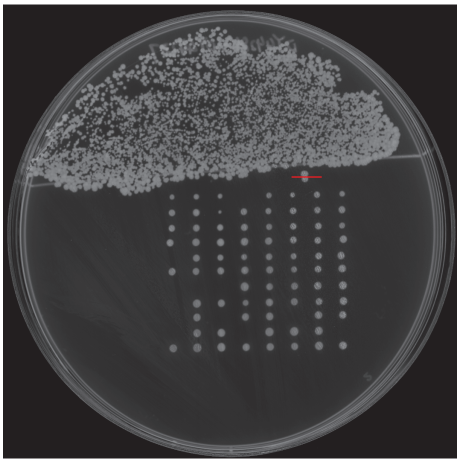

DY44286 × DY44287
2020-2
Successful octad: 11

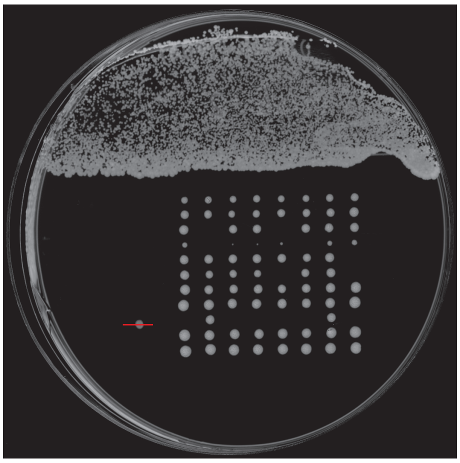

DY44286 × DY44287
2019-4
Successful octad: 10

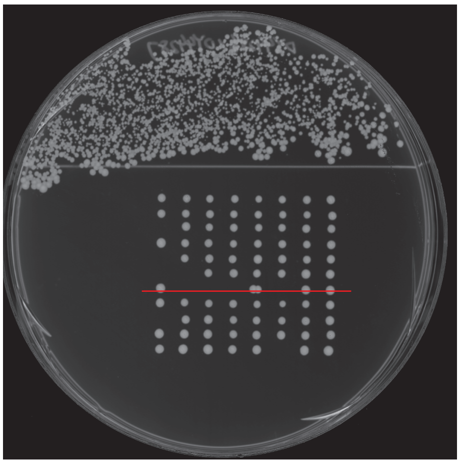

DY44286 × DY44287
2020-3
Successful octad: 11

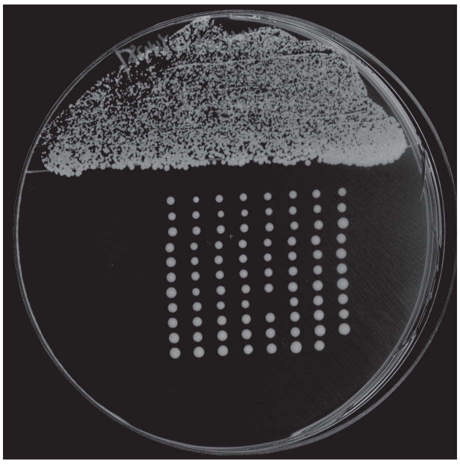

Wild type cross

YEST plate

DY44286 × DY44287
2021-1
Successful octad: 11

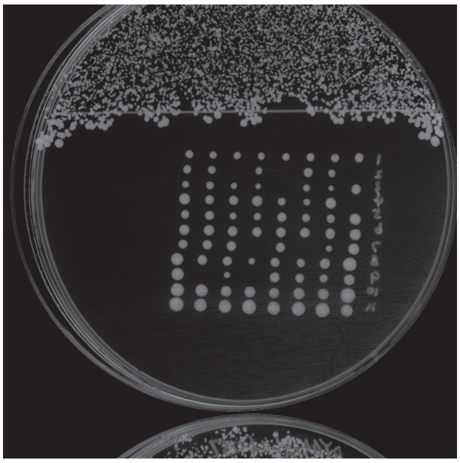

DY44286 × DY44287
2021-2
Successful octad: 11

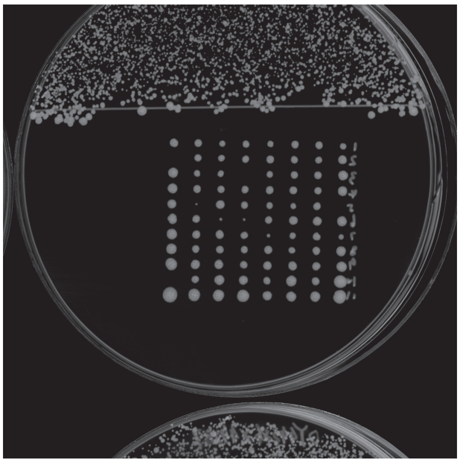

DY44286 × DY44287
2021-3
Successful octad: 11

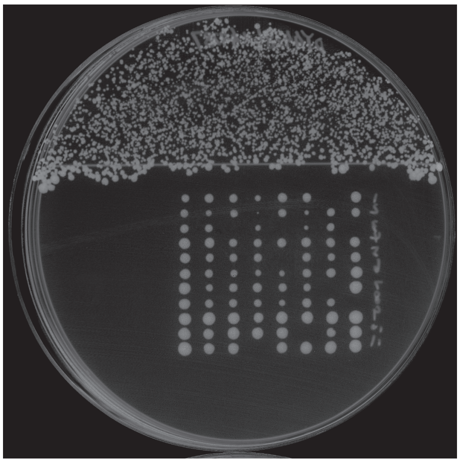

DY44286 × DY44287
2021-4
Successful octad: 11

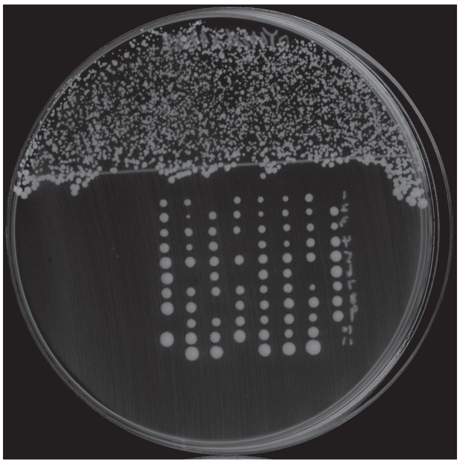

YEST plate

DY44286 × DY44287
2021-5
Successful octad: 10

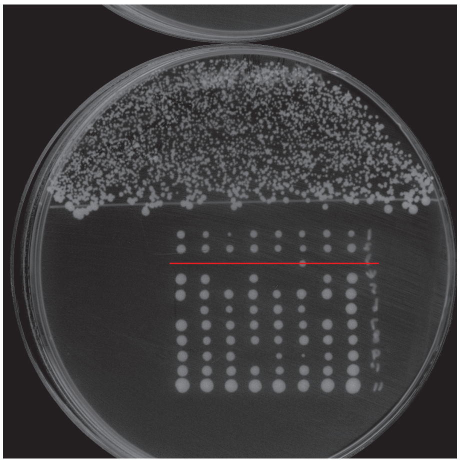

DY44286 × DY44287
2021-6
Successful octad: 11

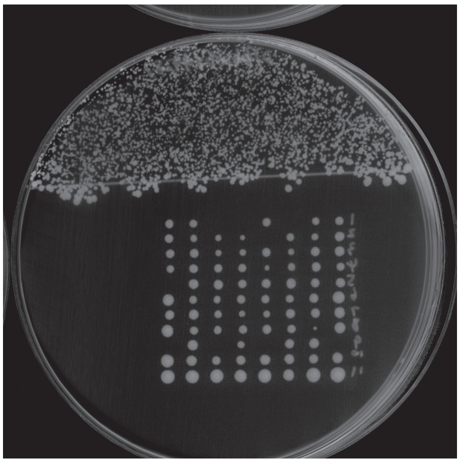

Supplement: Figure 8—source data 2. — Wild-type cross raw data files are shown as a pdf file with each cross in the upper left of the images. [file elife-81149-fig8-data2.pdf]
