# Supplementary material for: The wtf meiotic driver gene family has unexpectedly persisted for over 100 million years
Source: eLife. 2022 Oct 13;11:e81149. doi: 10.7554/eLife.81149 (PMC9562144; doi:10.7554/eLife.81149)

wtf25(SOCG_04480)Δ/wtf25(SOCG_04480)⁺ heterozygous diploid

	YEST plate	G418 plate

DY47905 cross-1

Successful octad: 10

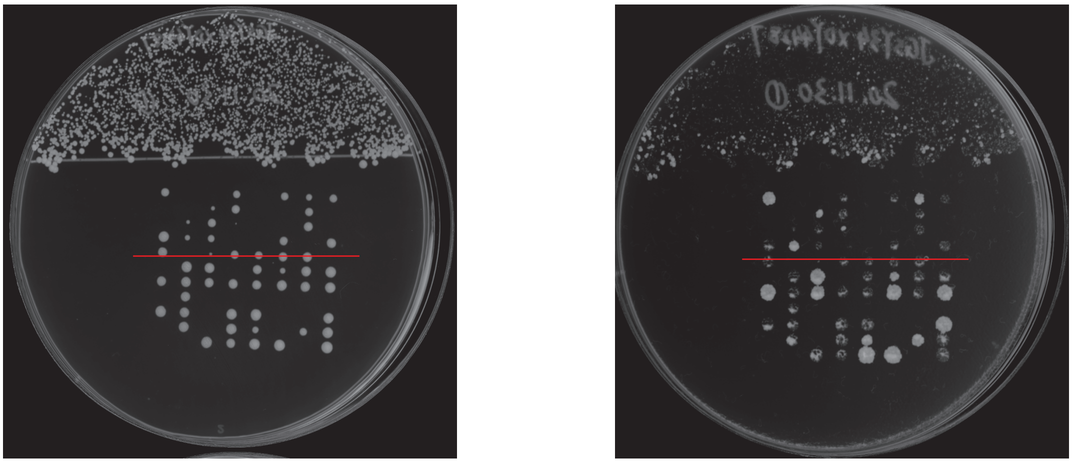

DY47905 cross-2

Successful octad: 11

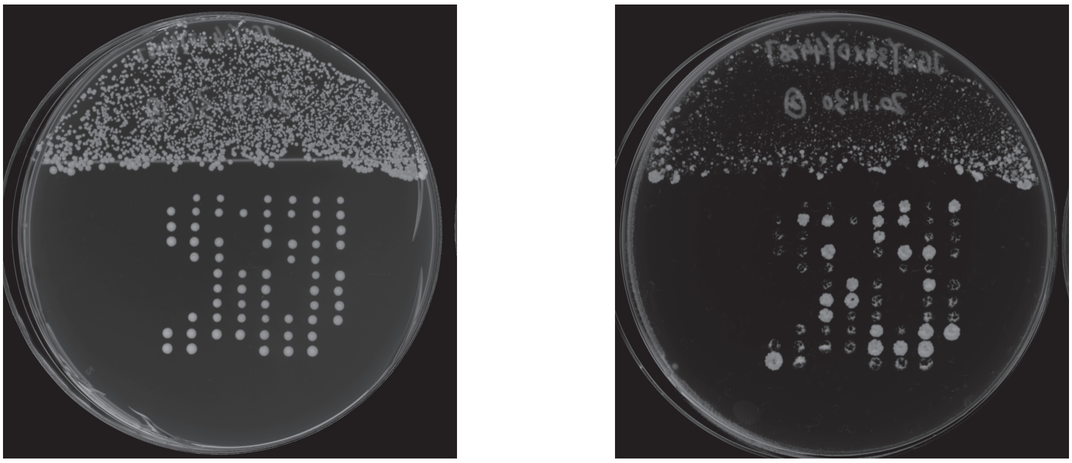

DY47905 cross-3

Successful octad: 10

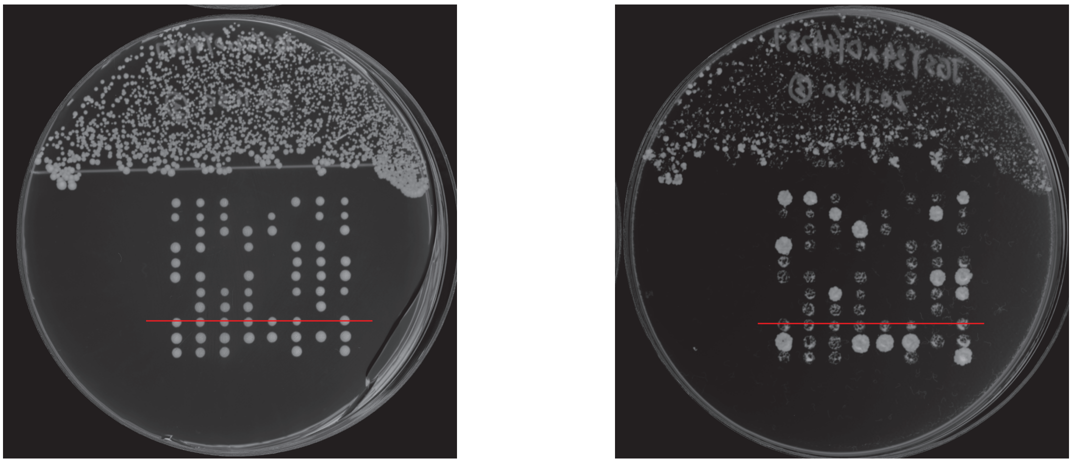

DY47905 cross-4

Successful octad: 5

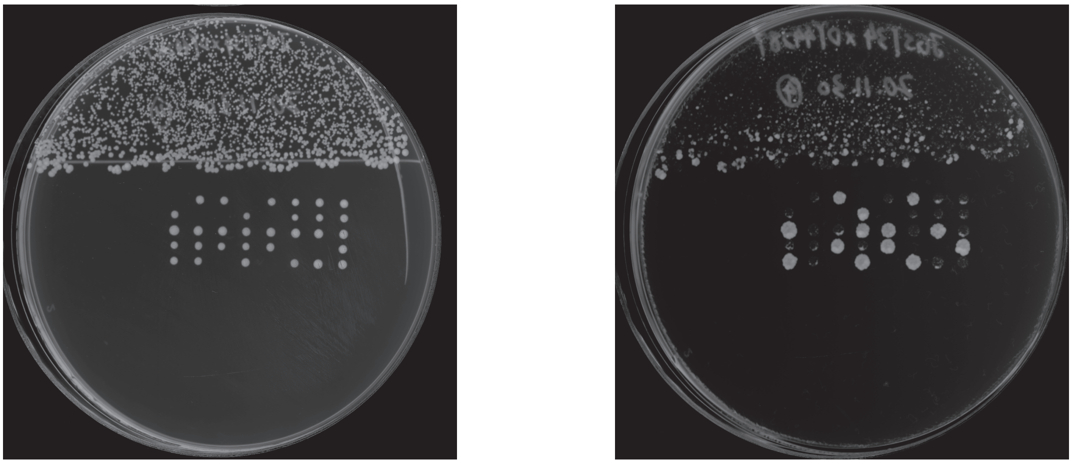

wtf25(SOCG_04480)Δ/wtf25(SOCG_04480)$^{+}$ heterozygous diploid

	YEST plate	G418 plate

DY47905 cross-5

Successful octad: 11

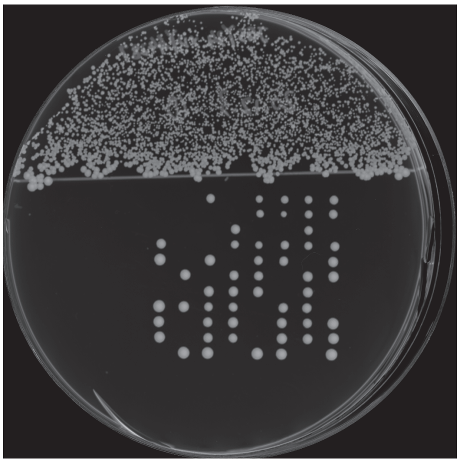

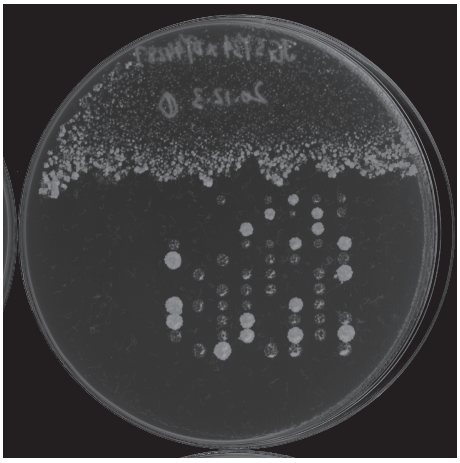

DY47905 cross-6

Successful octad: 11

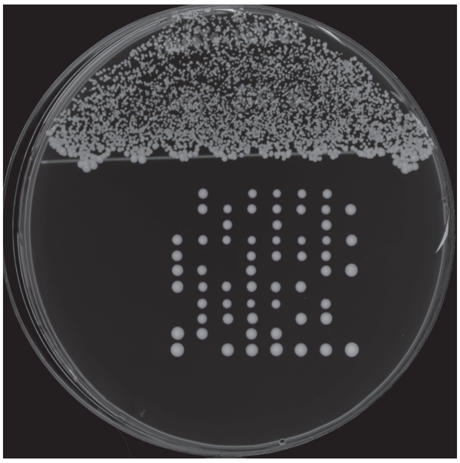

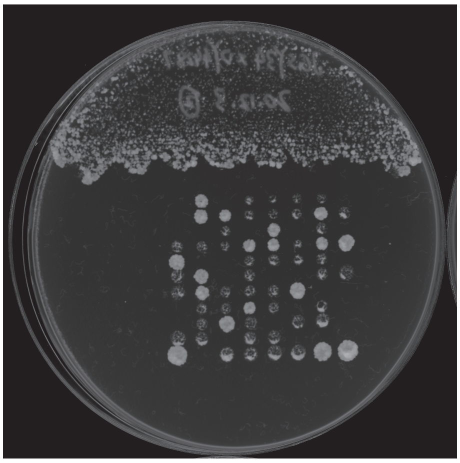

DY47905 cross-7

Successful octad: 11

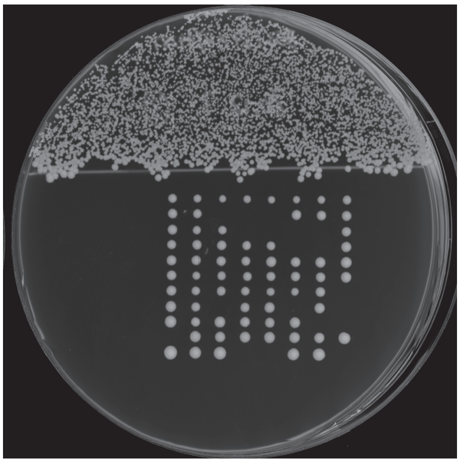

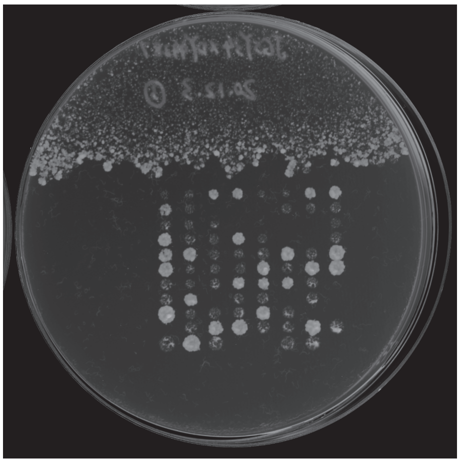

DY47905 cross-8

Successful octad: 10

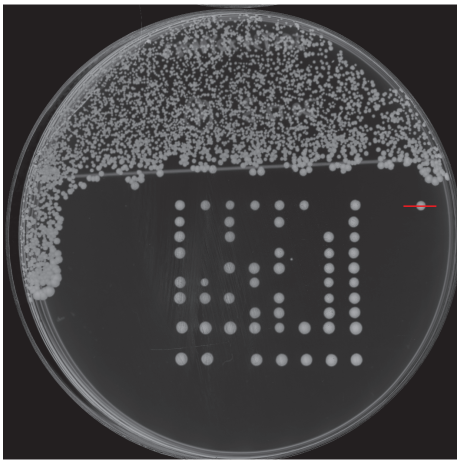

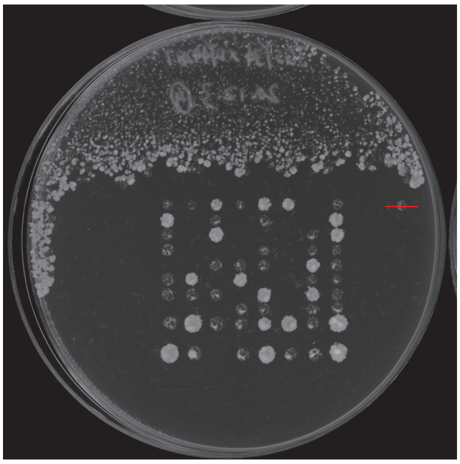

wtf25(SOCG_04480)Δ/wtf25(SOCG_04480)$^{+}$ heterozygous diploid

YEST plate	G418 plate

DY47906 cross-1

Successful octad: 11

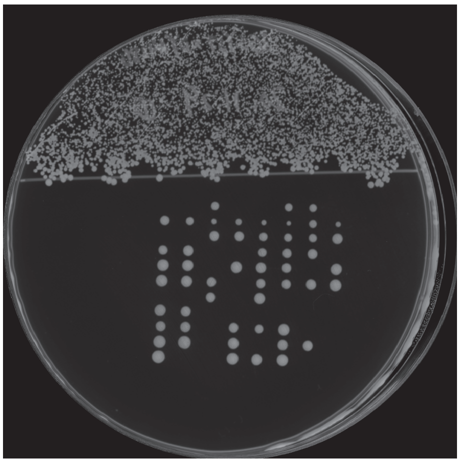

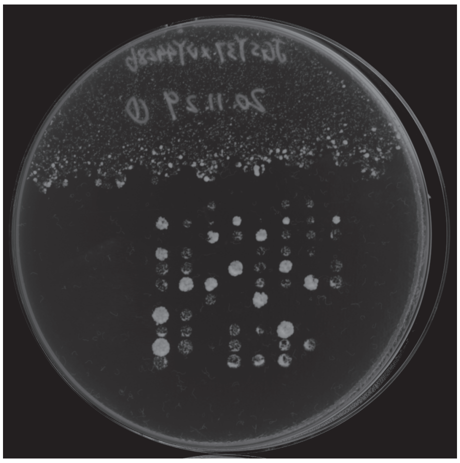

DY47906 cross-2

Successful octad: 11

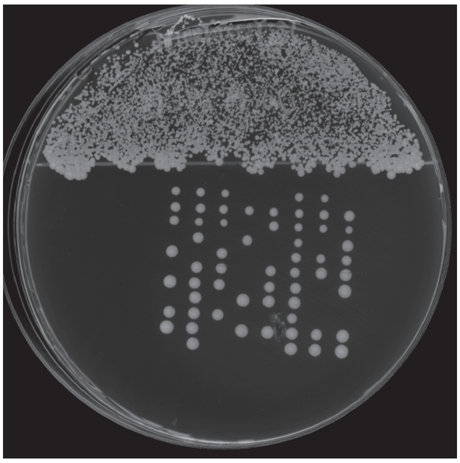

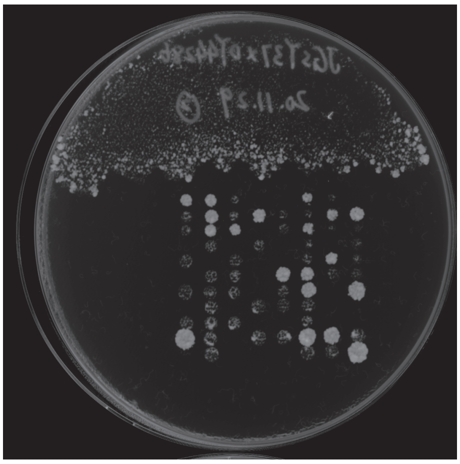

DY47906 cross-3

Successful octad: 10

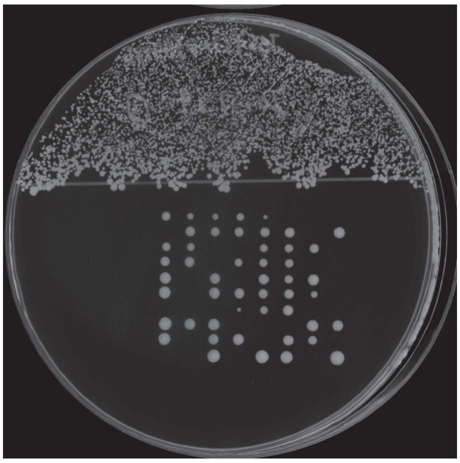

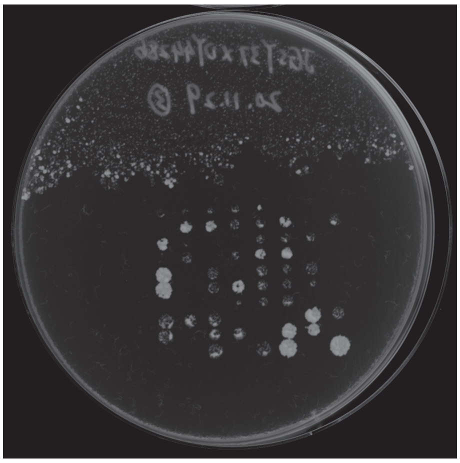

DY47906 cross-4

Successful octad: 7

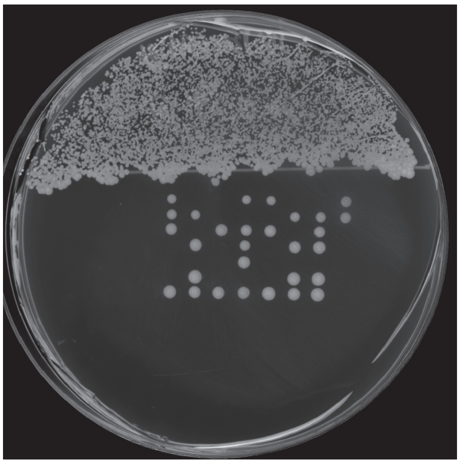

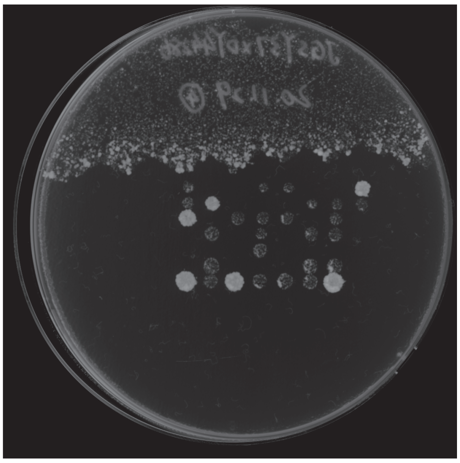

wtf25(SOCG_04480)Δ/wtf25(SOCG_04480)$^{+}$ heterozygous diploid

YEST plate | G418 plate

DY47906 cross-5

Successful octad: 11

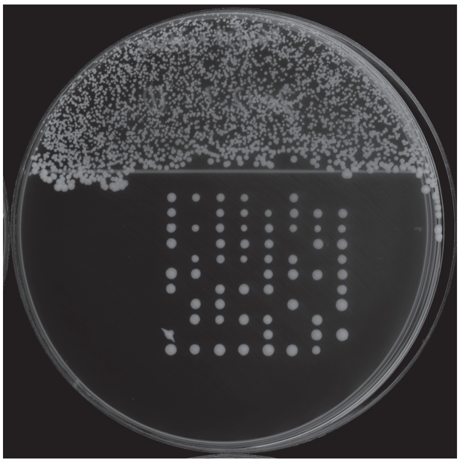

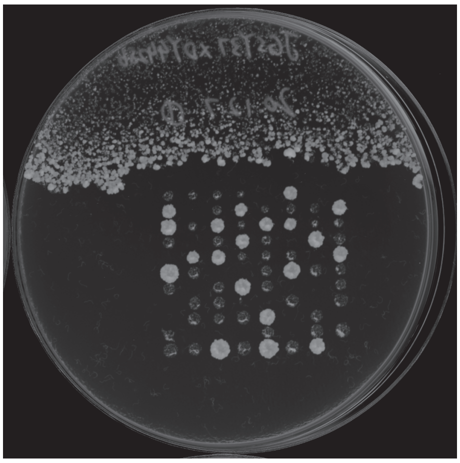

DY47906 cross-6

Successful octad: 11

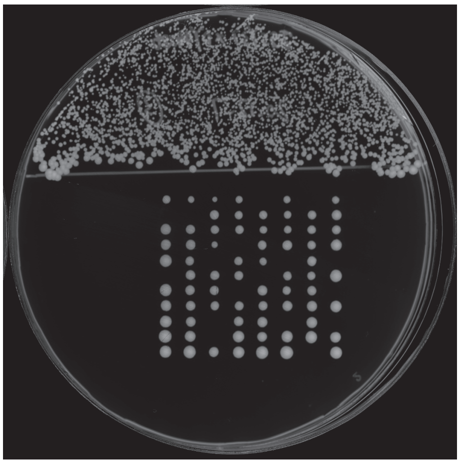

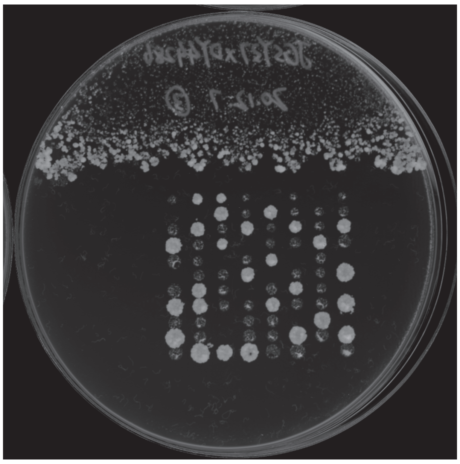

DY47906 cross-7

Successful octad: 11

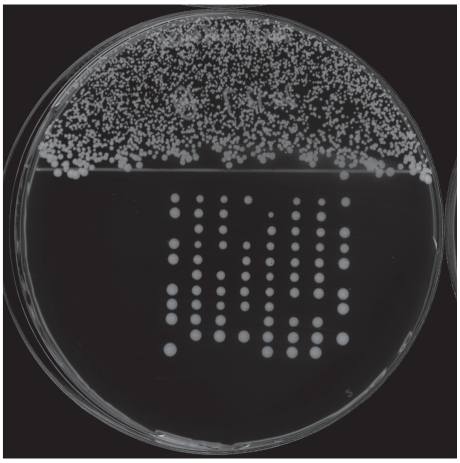

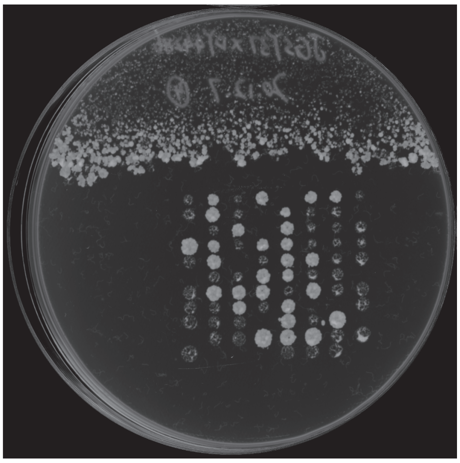

Supplement: Figure 9—source data 2. — wtf25+/wtf25Δ heterozygous diploid raw data files are shown as a pdf file with each cross in the upper left of the images. [file elife-81149-fig9-data2.pdf]
