# Supplementary material for: The wtf meiotic driver gene family has unexpectedly persisted for over 100 million years
Source: eLife. 2022 Oct 13;11:e81149. doi: 10.7554/eLife.81149 (PMC9562144; doi:10.7554/eLife.81149)
Supplement: Figure 9—source data 3. — wtf25Δ/wtf25Δ homozygous diploid raw data files are shown as a pdf file with each cross in the upper left of the images. [file elife-81149-fig9-data3.pdf]

wtf25(SOCG_04480)Δ/wtf25(SOCG_04480)Δ homozygous diploid

YEST plate | G418 plate

DY47905 × DY47906 -1

Successful octad: 11

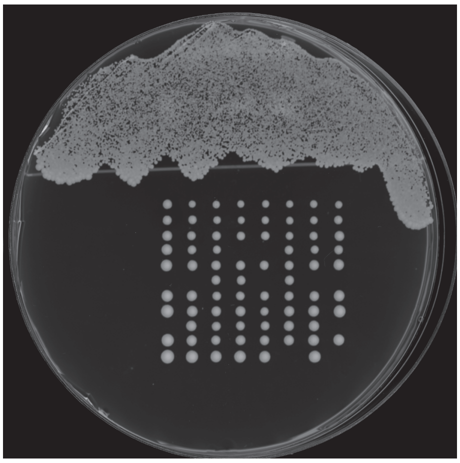

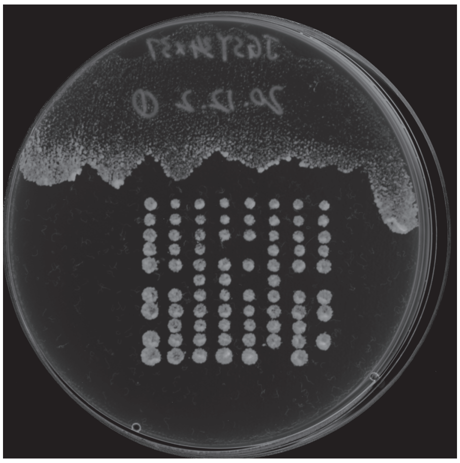

DY47905 × DY47906 -2

Successful octad: 11

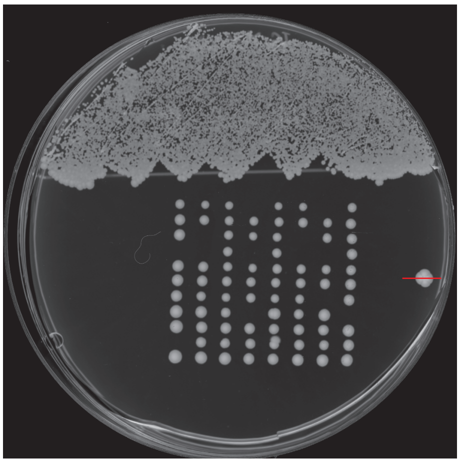

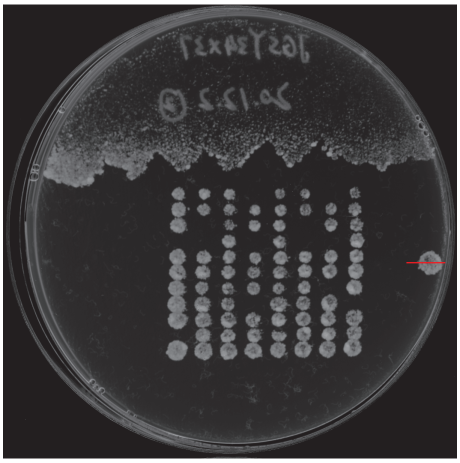

DY47905 × DY47906 -3

Successful octad: 10

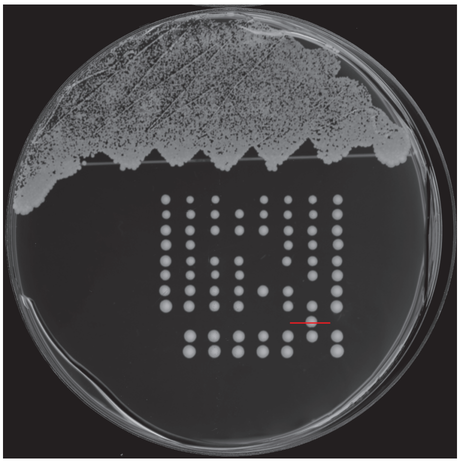

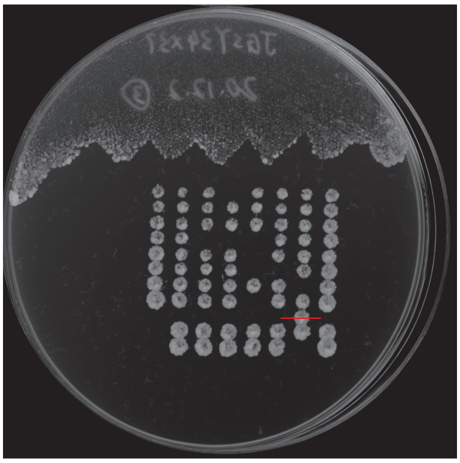

DY47905 × DY47906 -4

Successful octad: 10

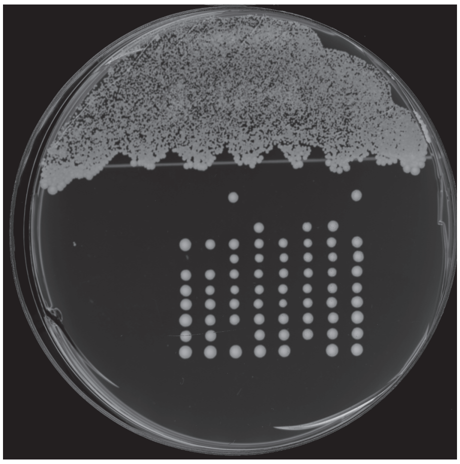

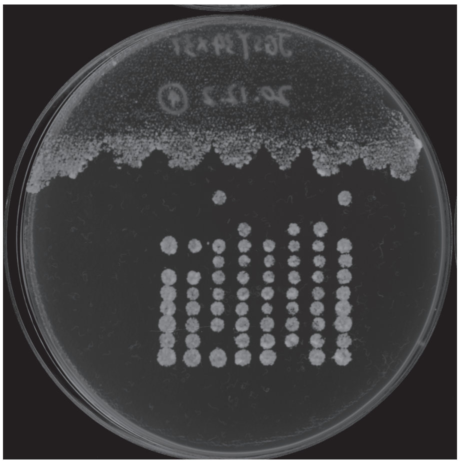

wtf25(SOCG_04480)Δ/wtf25(SOCG_04480)Δ homozygous diploid

	YEST plate	G418 plate
DY47905 × DY47906 -5<br>Successful octad: 11	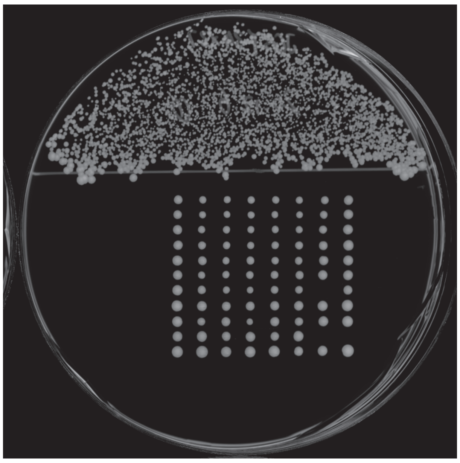	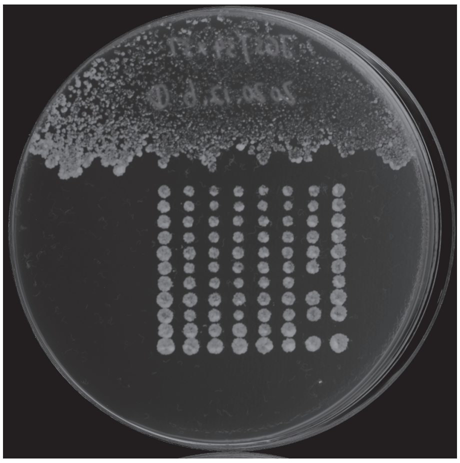
DY47905 × DY47906 -6<br>Successful octad: 11	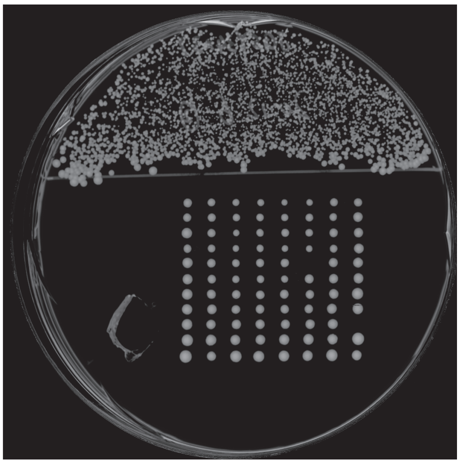	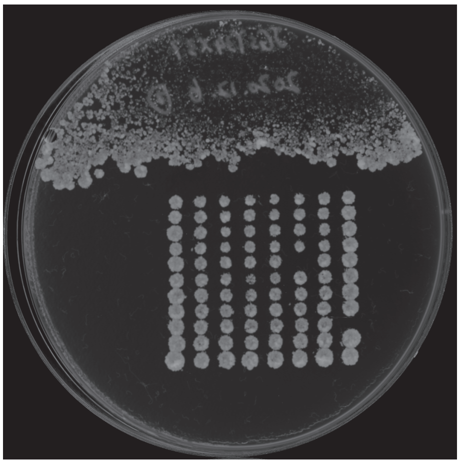
DY47905 × DY47906 -7<br>Successful octad: 11	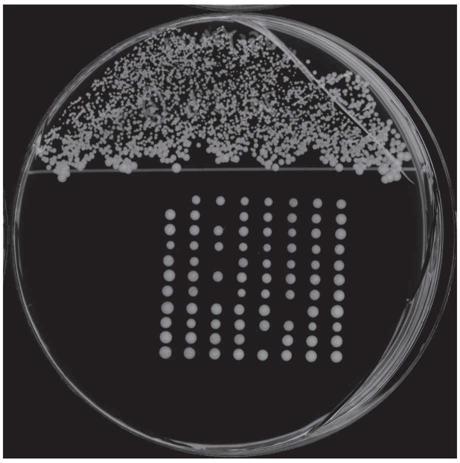	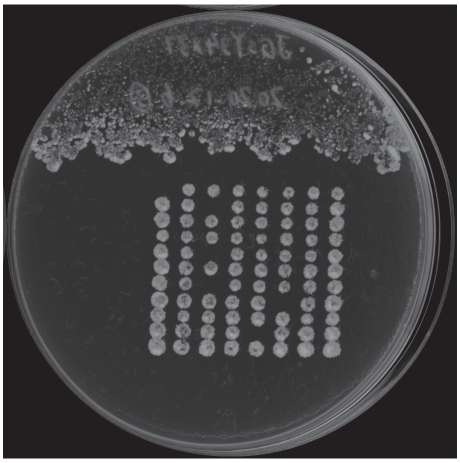
DY47905 × DY47906 -8<br>Successful octad: 10	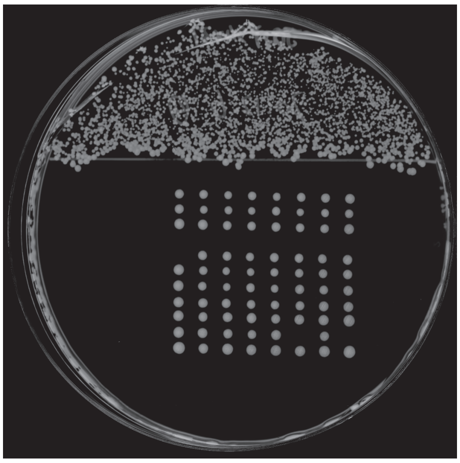	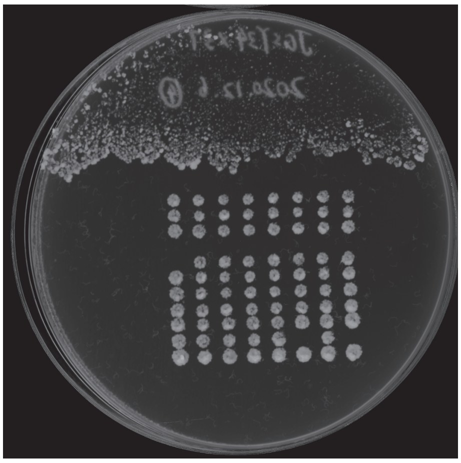
