# Supplementary material for: The wtf meiotic driver gene family has unexpectedly persisted for over 100 million years
Source: eLife. 2022 Oct 13;11:e81149. doi: 10.7554/eLife.81149 (PMC9562144; doi:10.7554/eLife.81149)

wtf68(SOCG_01240)Δ/wtf68(SOCG_01240)$^{+}$ heterozygous diploid

	YEST plate	G418 plate
DY47919 cross-1<br>Successful octad: 11	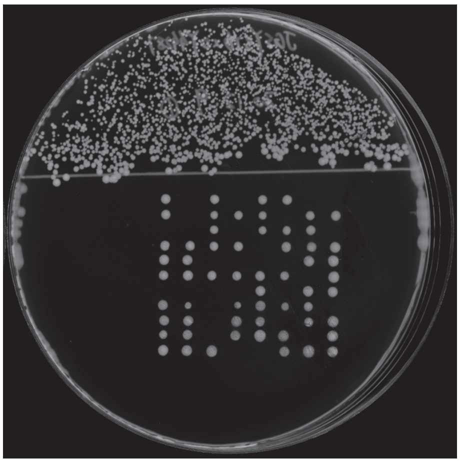	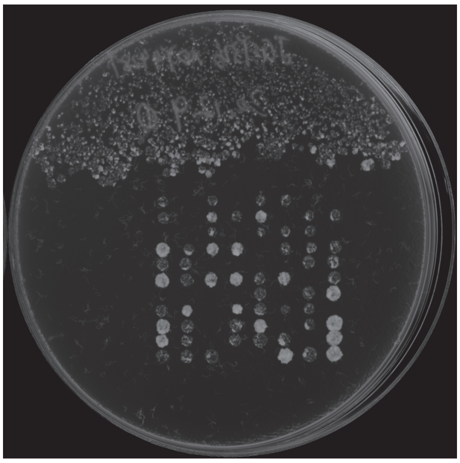
DY47919 cross-2<br>Successful octad: 9	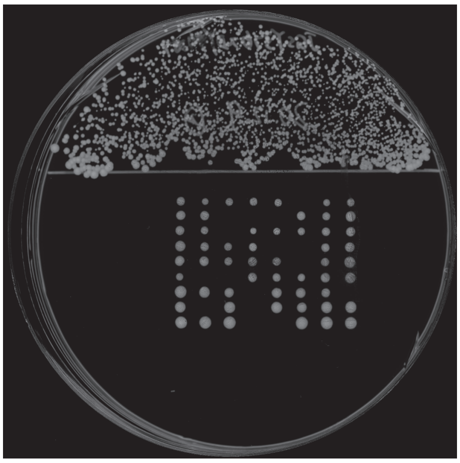	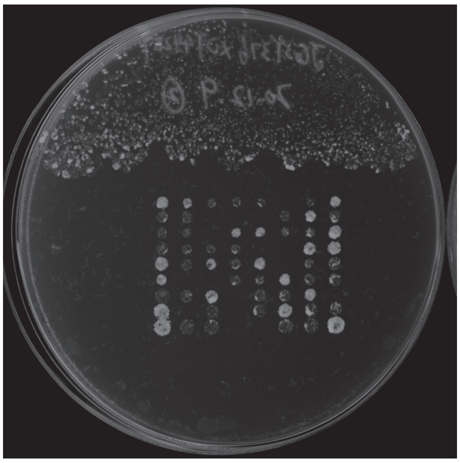
DY47919 cross-3<br>Successful octad: 11	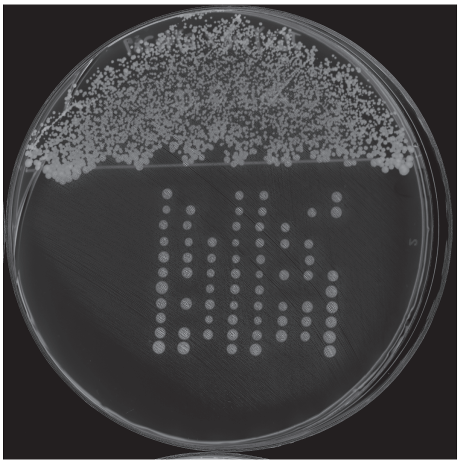	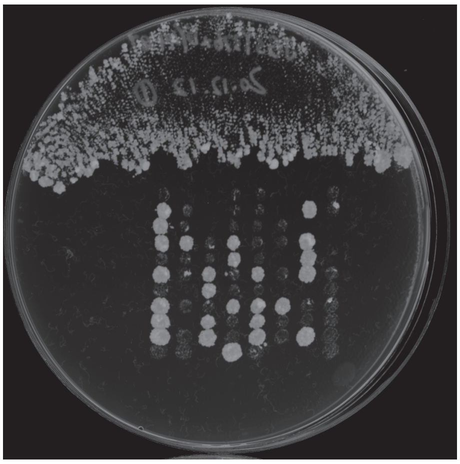
DY47919 cross-4<br>Successful octad: 11	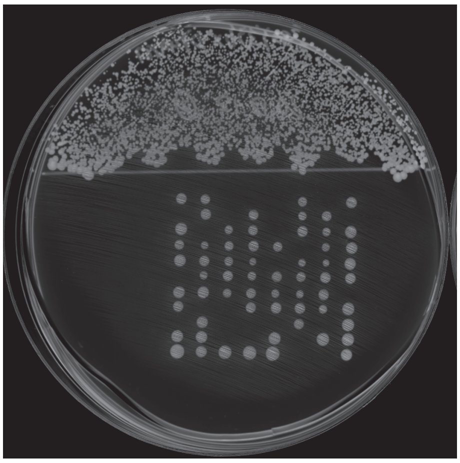	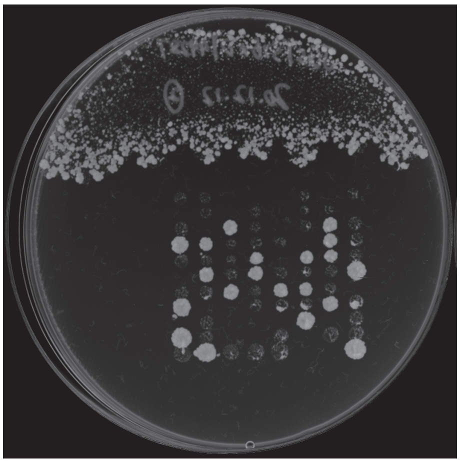

wtf68(SOCG_01240)Δ/wtf68(SOCG_01240)$^{+}$ heterozygous diploid

DY47919 cross-5

Successful octad: 11

YEST plate

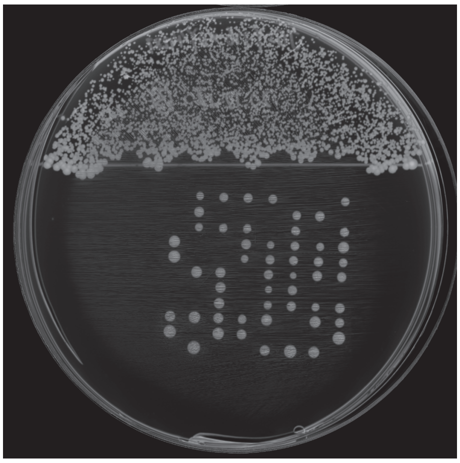

G418 plate

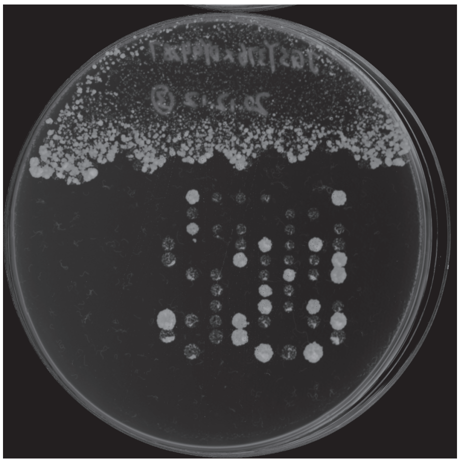

wtf68(SOCG_01240)Δ/wtf68(SOCG_01240)$^{+}$ heterozygous diploid

YEST plate | G418 plate

DY47920 cross-1

Successful octad: 11

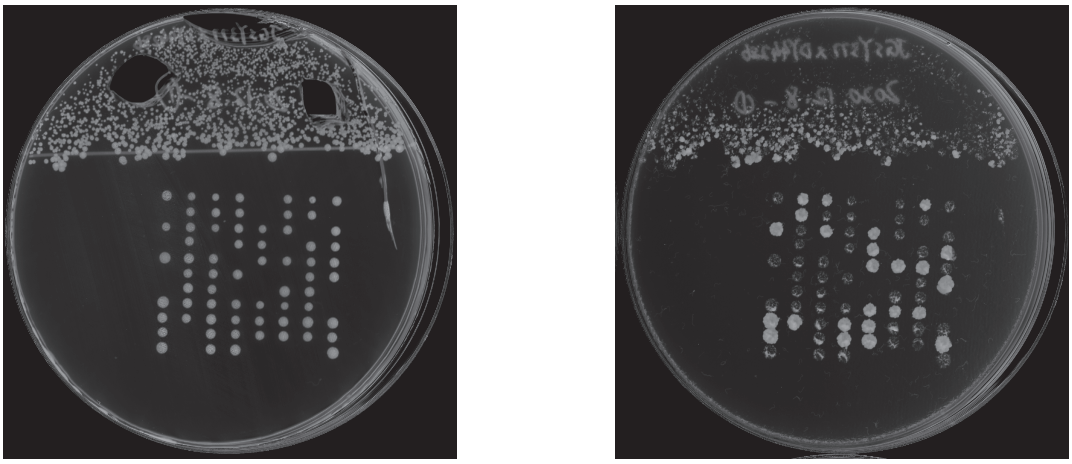

DY47920 cross-2

Successful octad: 10

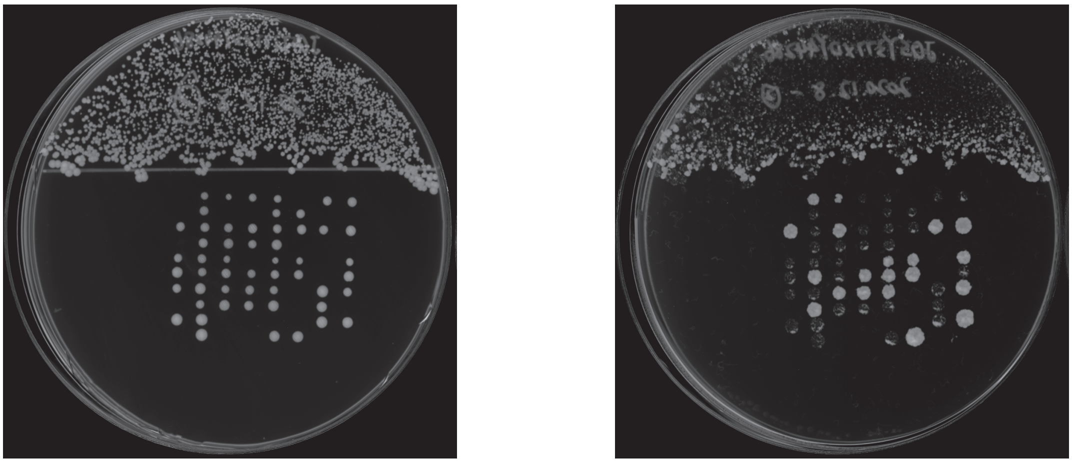

DY47920 cross-3

Successful octad: 11

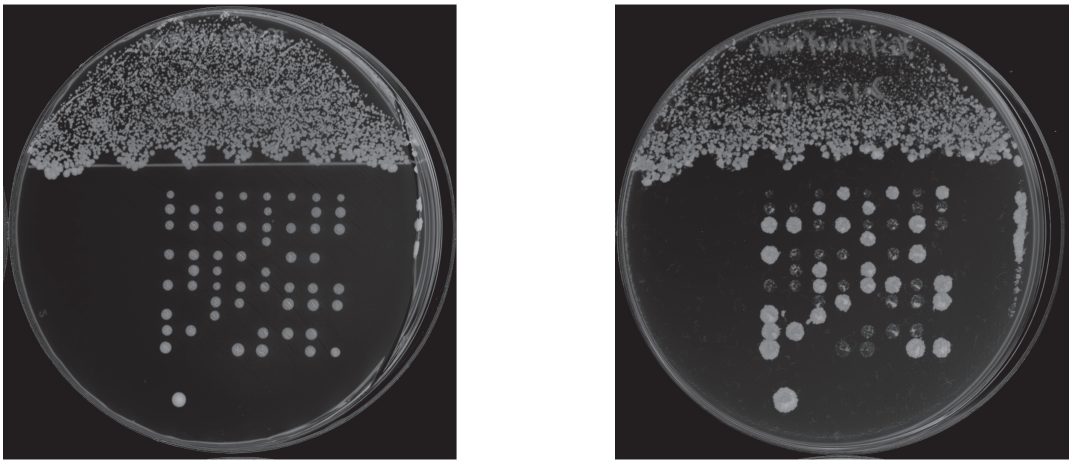

DY47920 cross-4

Successful octad: 11

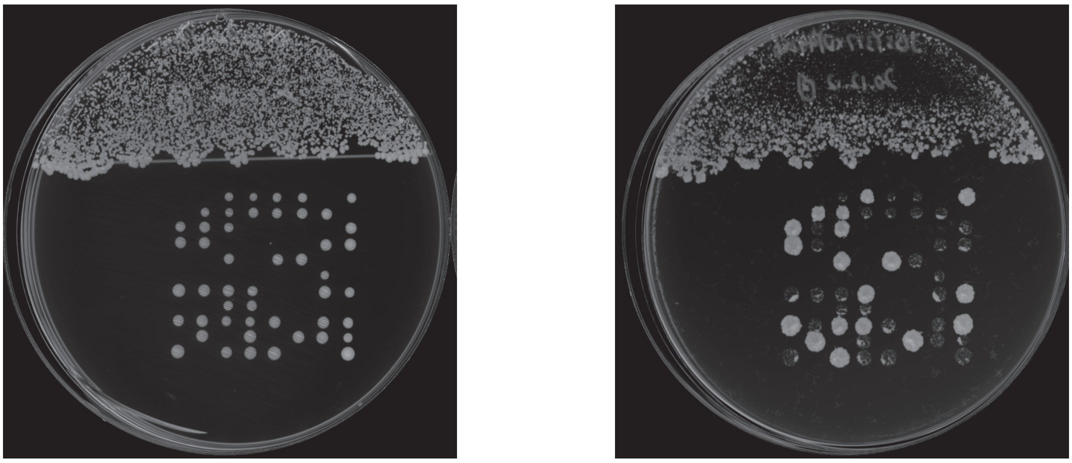

wtf68(SOCG_01240)Δ/wtf68(SOCG_01240)$^{+}$ heterozygous diploid

DY47920 cross-5

Successful octad: 11

YEST plate

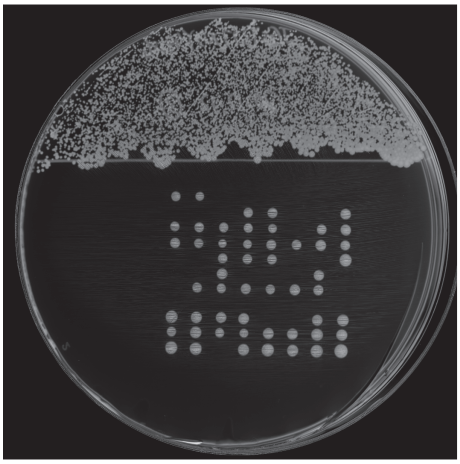

G418 plate

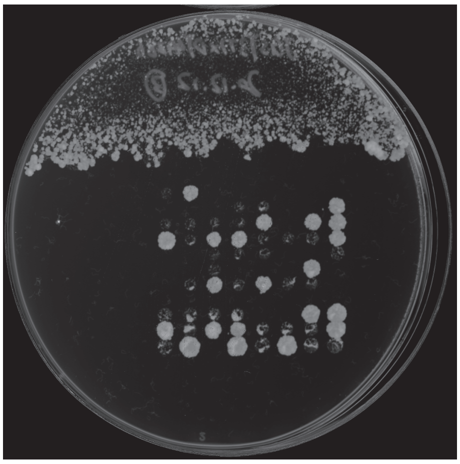

Supplement: Figure 9—figure supplement 1—source data 2. — wtf68+/wtf68Δ heterozygous diploid raw data files are shown as a pdf file with each cross in the upper left of the images. [file elife-81149-fig9-figsupp1-data2.pdf]
