# Supplementary material for: The wtf meiotic driver gene family has unexpectedly persisted for over 100 million years
Source: eLife. 2022 Oct 13;11:e81149. doi: 10.7554/eLife.81149 (PMC9562144; doi:10.7554/eLife.81149)

wtf68(SOCG_01240)Δ/wtf68(SOCG_01240)Δ homozygous diploid

	YEST plate	G418 plate
DY47919 × DY47920 -1<br>Successful octad: 11	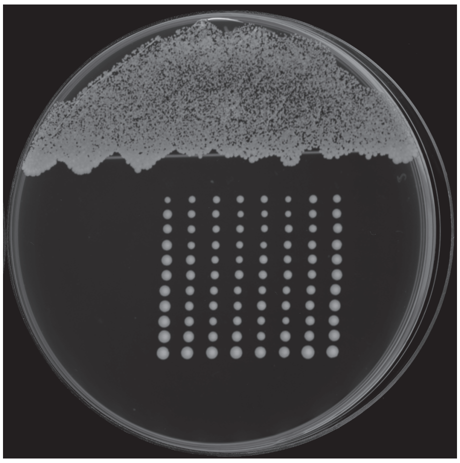	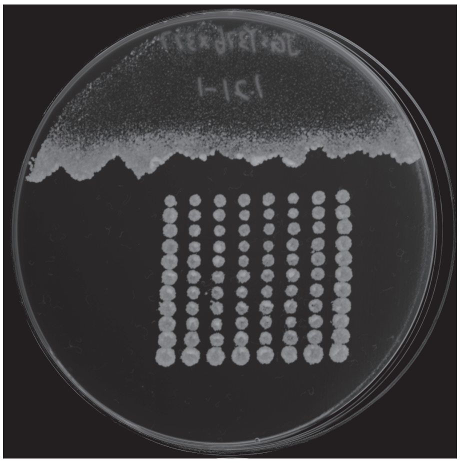
DY47919 × DY47920 -2<br>Successful octad: 11	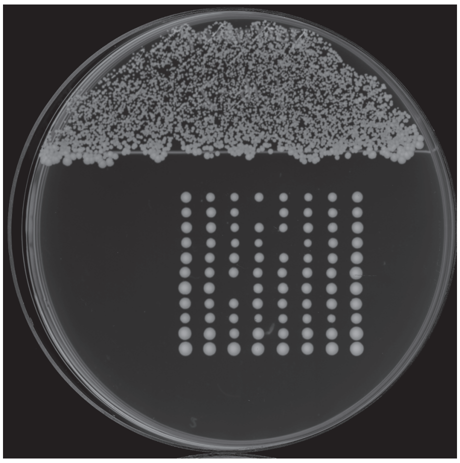	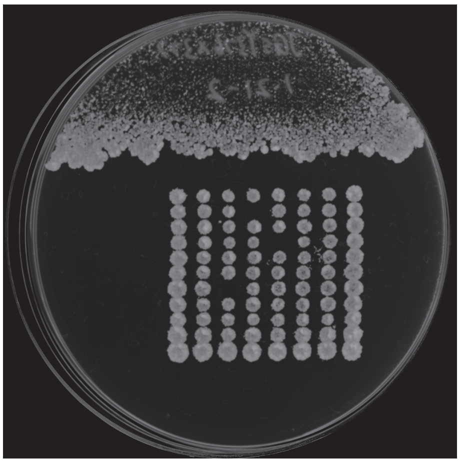
DY47919 × DY47920 -3<br>Successful octad: 11	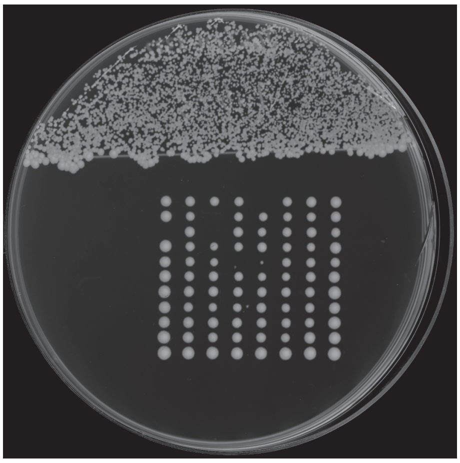	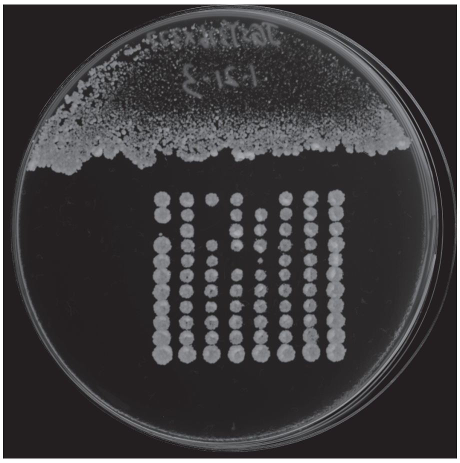
DY47919 × DY47920 -4<br>Successful octad: 11	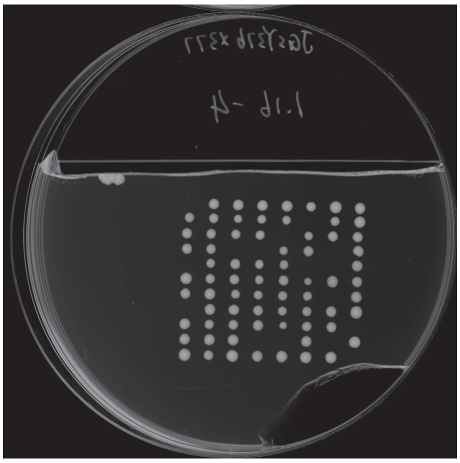	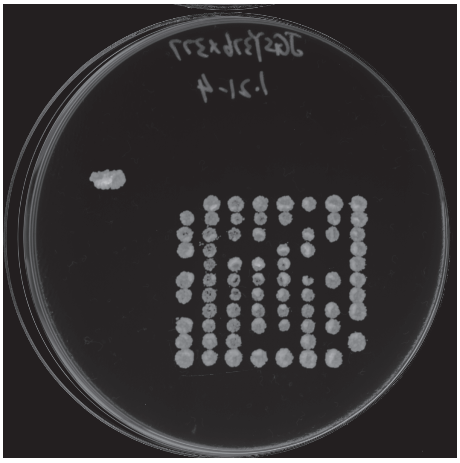

wtf68(SOCG_01240)Δ/wtf68(SOCG_01240)Δ homozygous diploid

YEST plate | G418 plate

DY47919 × DY47920 -5

Successful octad: 11

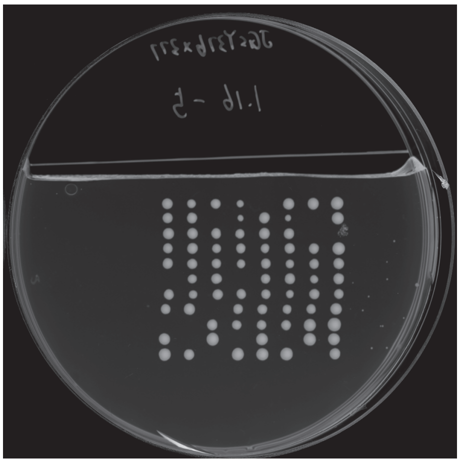

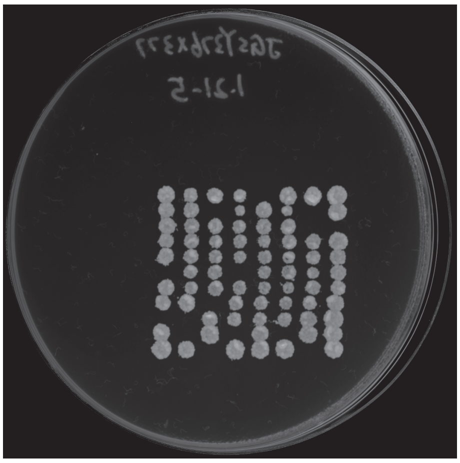

DY47919 × DY47920 -6

Successful octad: 11

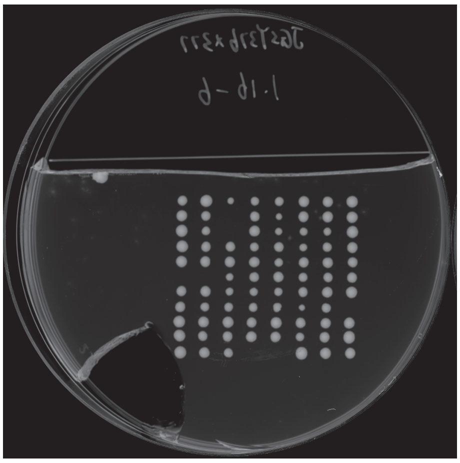

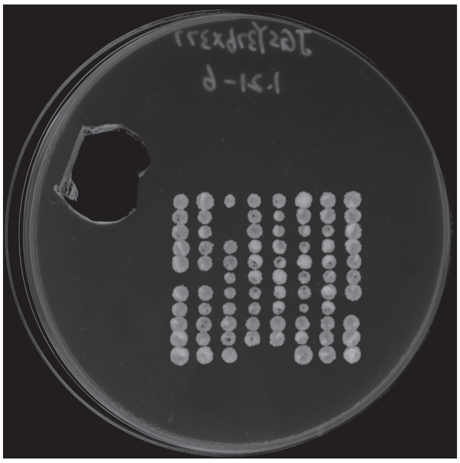

Supplement: Figure 9—figure supplement 1—source data 3. — wtf68Δ/wtf68Δ homozygous diploid raw data files are shown as a pdf file with each cross in the upper left of the images. [file elife-81149-fig9-figsupp1-data3.pdf]
