# Supplementary material for: The wtf meiotic driver gene family has unexpectedly persisted for over 100 million years
Source: eLife. 2022 Oct 13;11:e81149. doi: 10.7554/eLife.81149 (PMC9562144; doi:10.7554/eLife.81149)

wtf33Δ/wtf33⁺ heterozygous diploid

YEST plate | G418 plate

DY47903 cross-1

Successful octad: 10

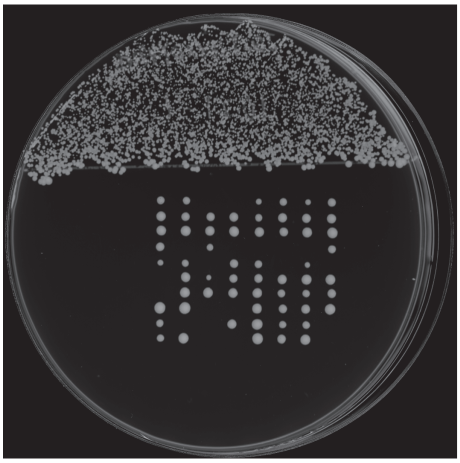

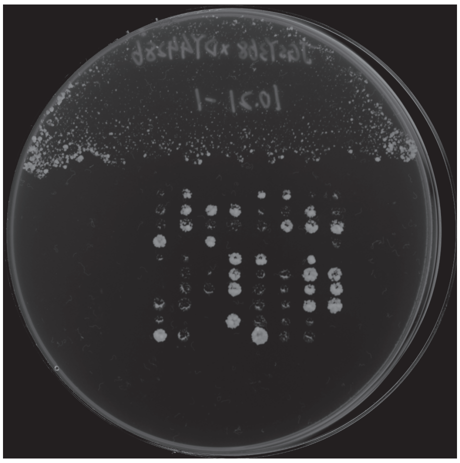

DY47903 cross-2

Successful octad: 11

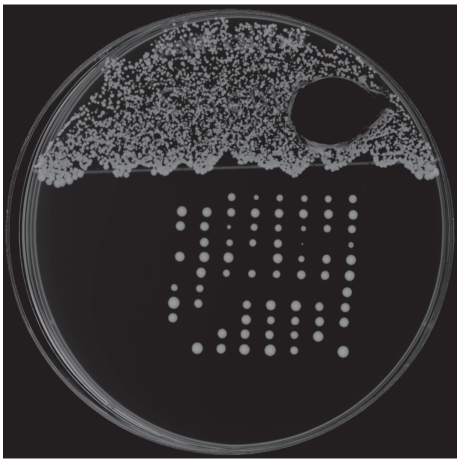

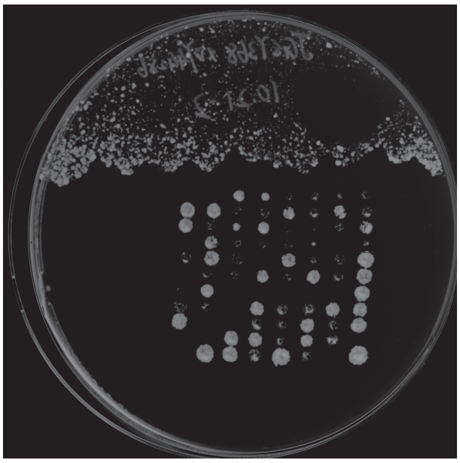

DY47903 cross-3

Successful octad: 10

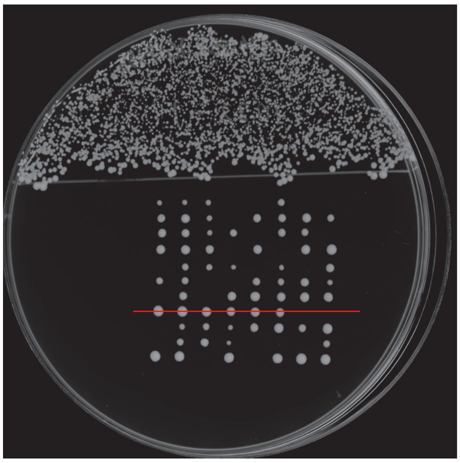

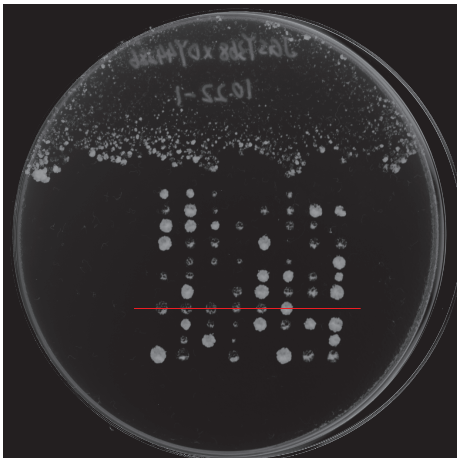

DY47903 cross-4

Successful octad: 11

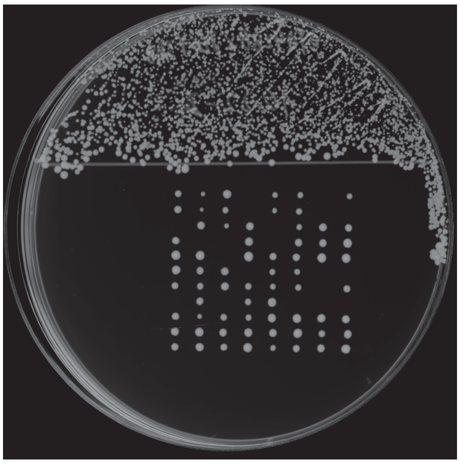

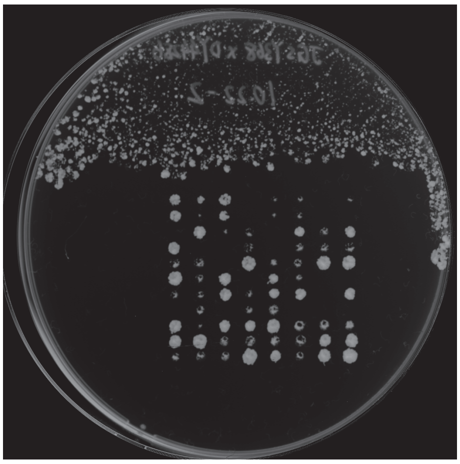

wtf33Δ/wtf33$^+$ heterozygous diploid

DY47903 cross-5

Successful octad: 11

YEST plate

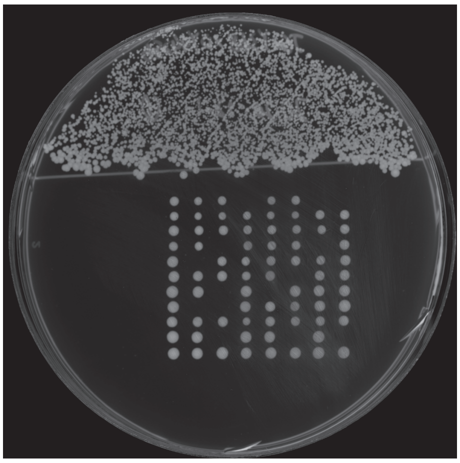

G418 plate

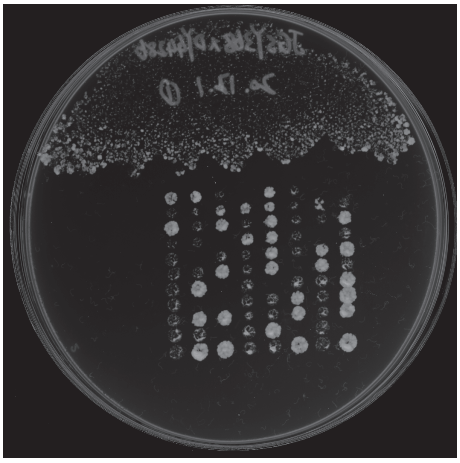

wtf33Δ/wtf33⁺ heterozygous diploid

YEST plate

G418 plate

DY47904 cross-1
Successful octad: 11

DY47904 cross-2
Successful octad: 11

DY47904 cross-3
Successful octad: 11

DY47904 cross-4
Successful octad: 11

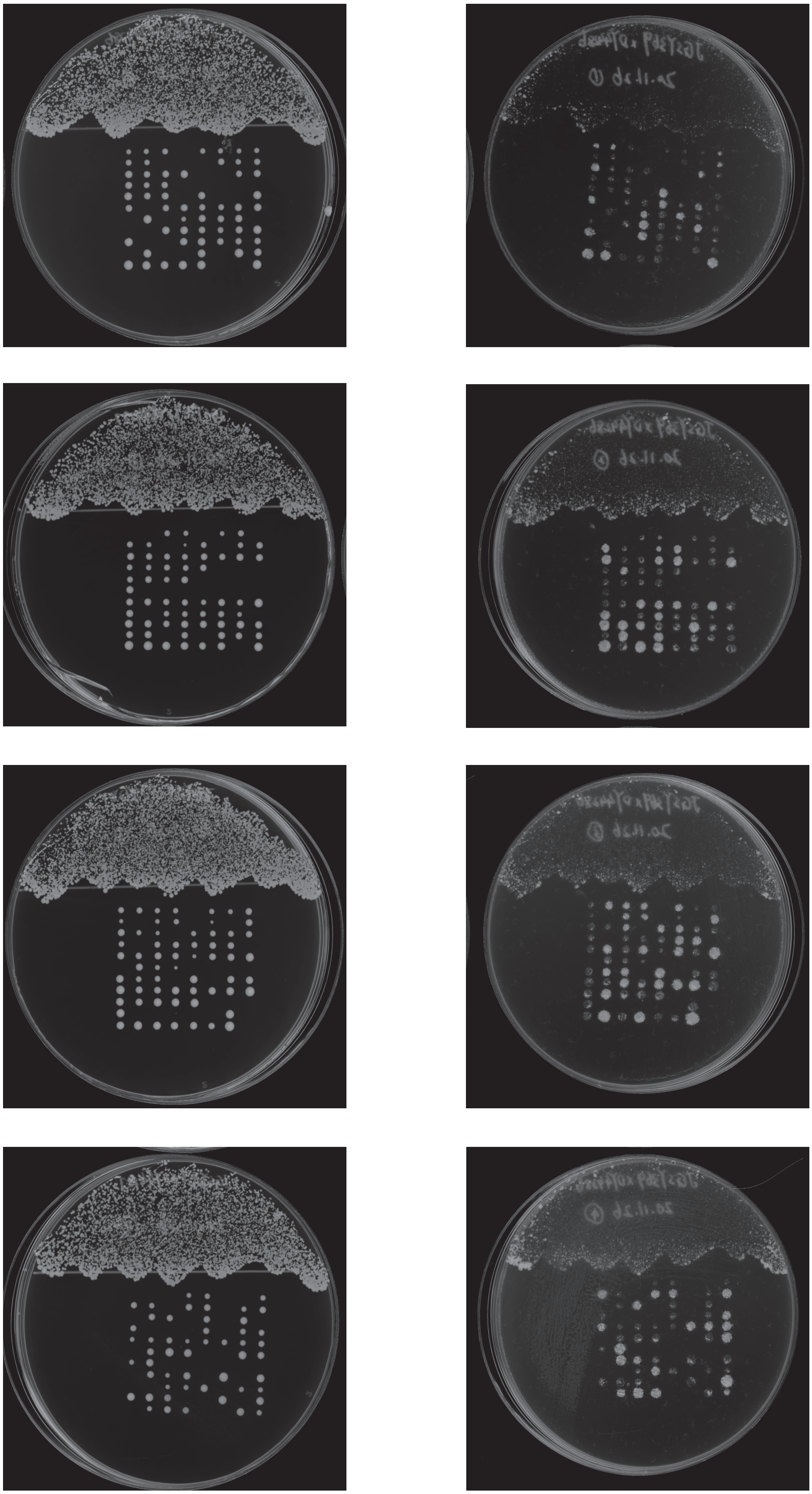

wtf33Δ/wtf33⁺ heterozygous diploid

DY47904 cross-5

Successful octad: 11

YEST plate

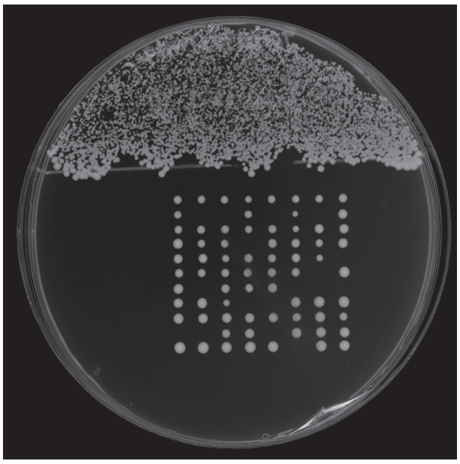

G418 plate

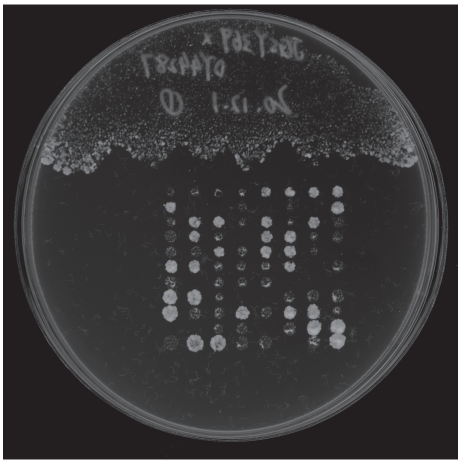

Supplement: Figure 9—figure supplement 2—source data 2. — wtf33+/wtf33Δ heterozygous diploid raw data files are shown as a pdf file with each cross in the upper left of the images. [file elife-81149-fig9-figsupp2-data2.pdf]
