# Supplementary material for: The wtf meiotic driver gene family has unexpectedly persisted for over 100 million years
Source: eLife. 2022 Oct 13;11:e81149. doi: 10.7554/eLife.81149 (PMC9562144; doi:10.7554/eLife.81149)

*wtf33Δ/wtf33Δ* homozygous diploid

| | YEST plate | G418 plate |
|---|---|---|
| DY47903 × DY47904 -1<br>Successful octad: 10 | 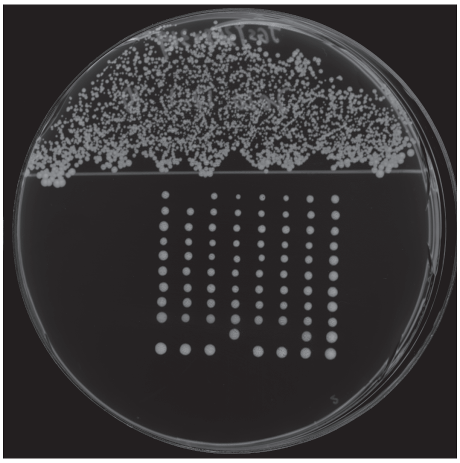 | 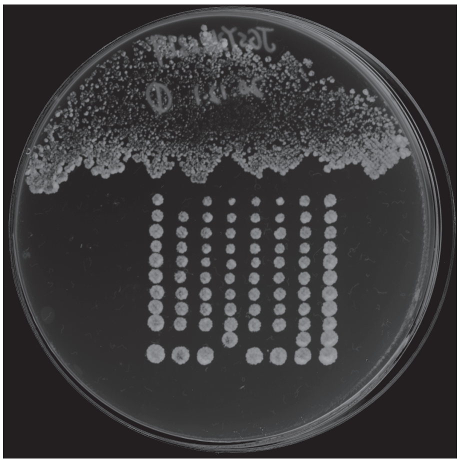 |
| DY47903 × DY47904 -2<br>Successful octad: 11 | 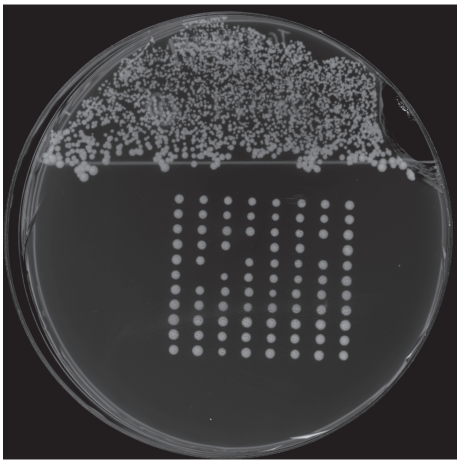 | 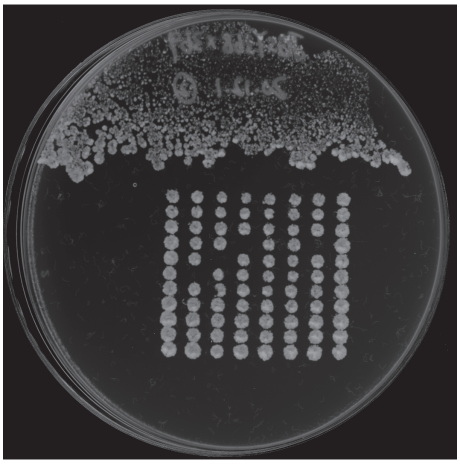 |
| DY47903 × DY47904 -3<br>Successful octad: 11 | 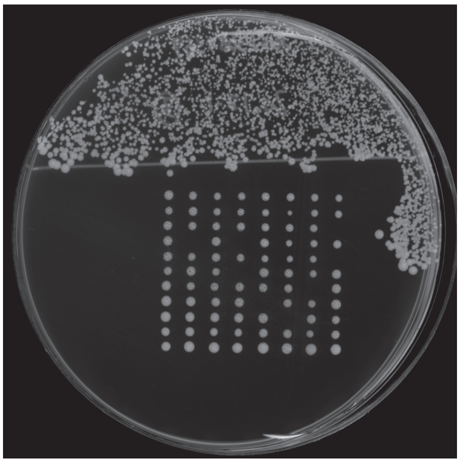 | 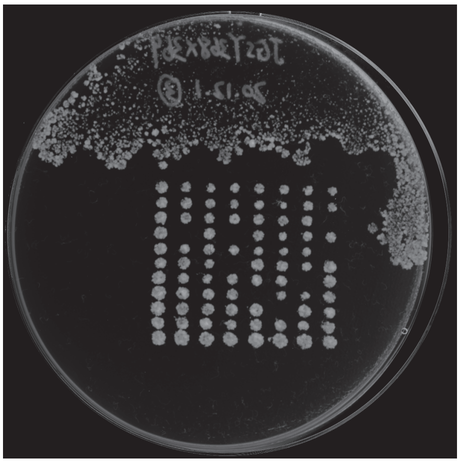 |
| DY47903 × DY47904 -4<br>Successful octad: 11 | 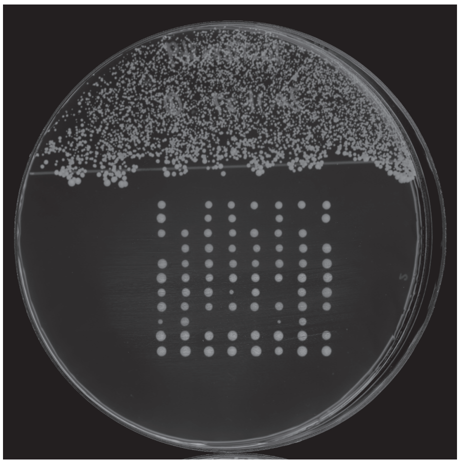 | 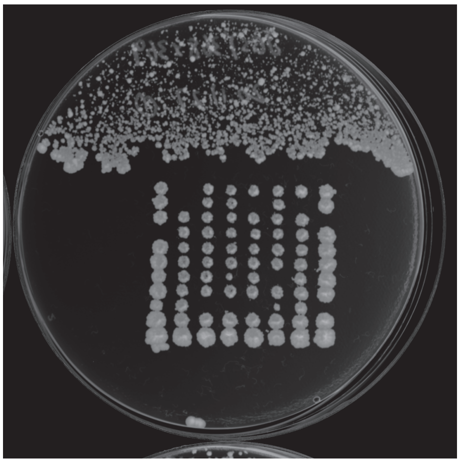 |

*wtf33Δ/wtf33Δ* homozygous diploid

YEST plate

G418 plate

DY47903 × DY47904 -5

Successful octad: 11

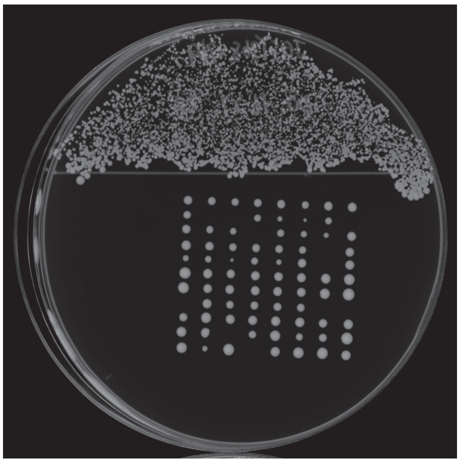

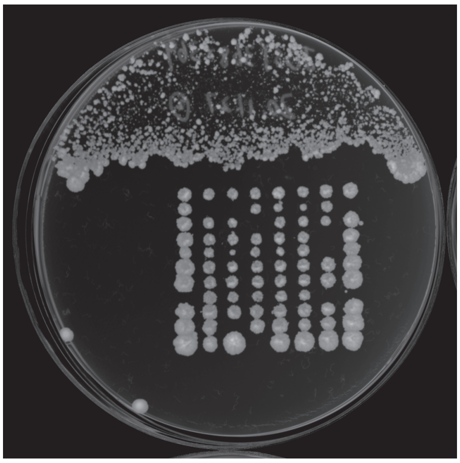

DY47903 × DY47904 -6

Successful octad: 10

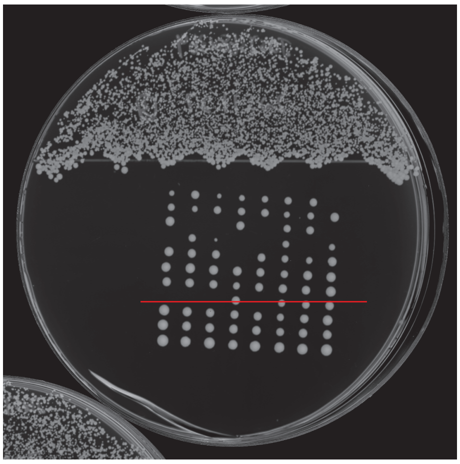

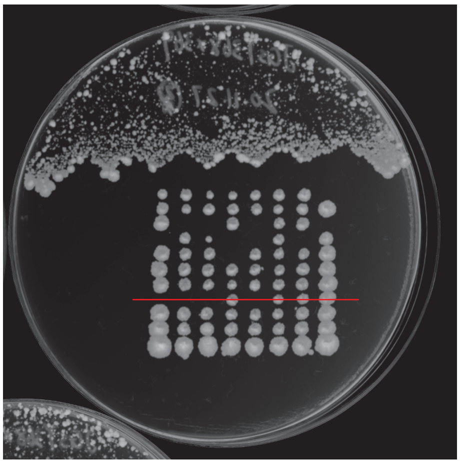

Supplement: Figure 9—figure supplement 2—source data 3. — wtf33Δ/wtf33Δ homozygous diploid raw data files are shown as a pdf file with each cross in the upper left of the images. [file elife-81149-fig9-figsupp2-data3.pdf]
