# Supplementary material for: The wtf meiotic driver gene family has unexpectedly persisted for over 100 million years
Source: eLife. 2022 Oct 13;11:e81149. doi: 10.7554/eLife.81149 (PMC9562144; doi:10.7554/eLife.81149)

wtf60(SOCG_04742)Δ/wtf60(SOCG_04742)$^{+}$ heterozygous diploid

YEST plate | G418 plate

DY47921 cross-1

Successful octad: 11

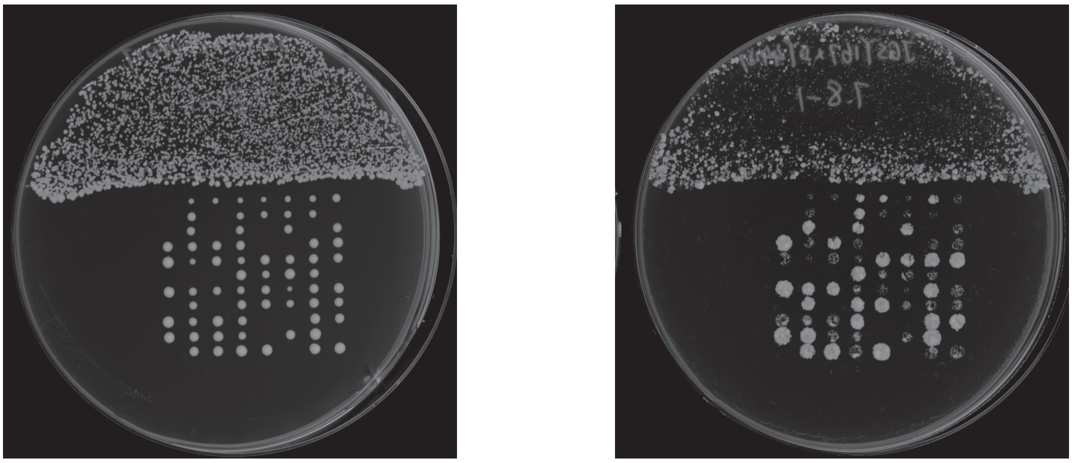

DY47921 cross-2

Successful octad: 11

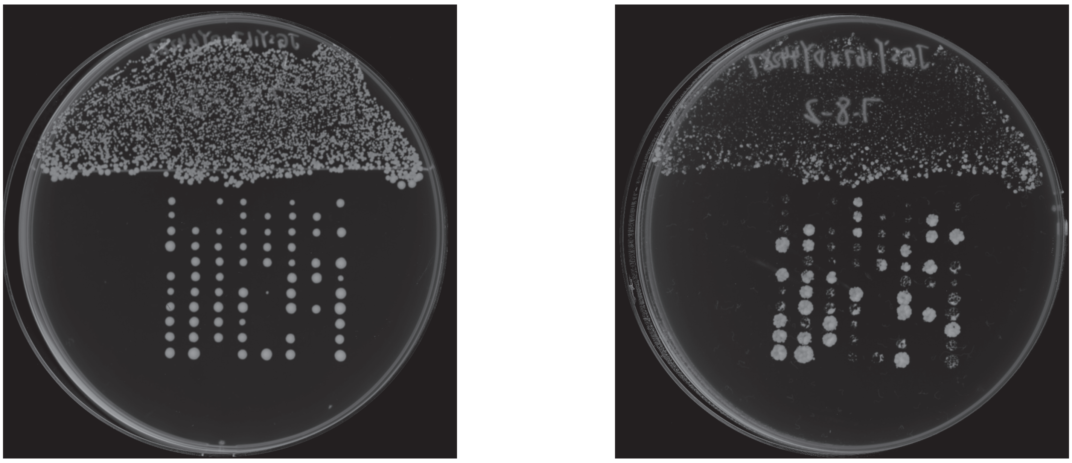

DY47921 cross-3

Successful octad: 10

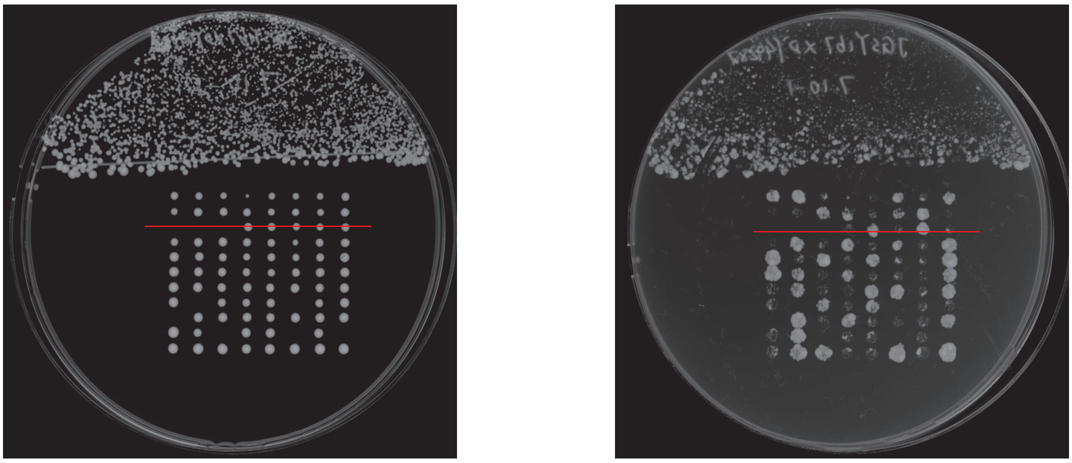

DY47921 cross-4

Successful octad: 3

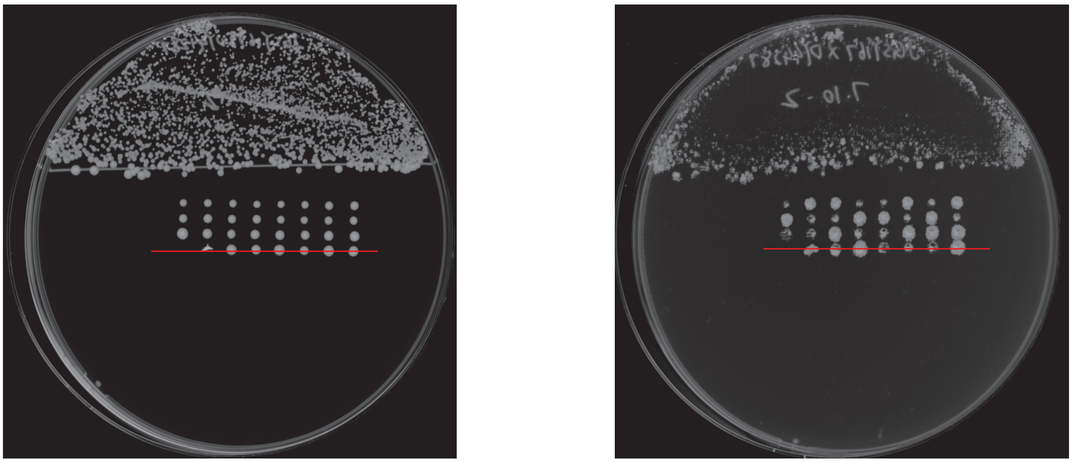

wtf60(SOCG_04742)Δ/wtf60(SOCG_04742)⁺ heterozygous diploid

YEST plate

G418 plate

DY47921 cross-5

Successful octad: 10

DY47921 cross-6

Successful octad: 11

DY47921 cross-7

Successful octad: 10

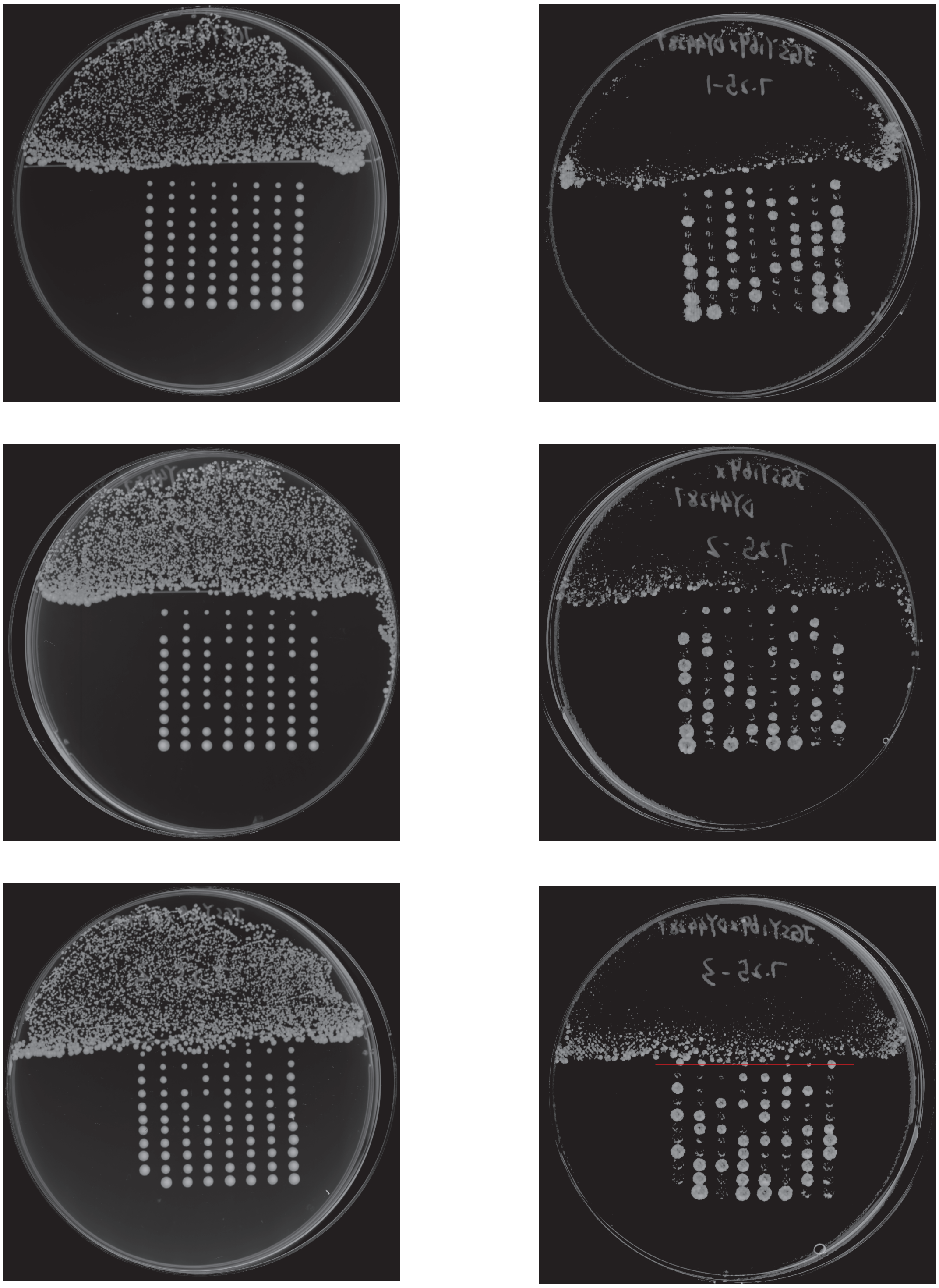

wtf60(SOCG_04742)Δ/wtf60(SOCG_04742)⁺ heterozygous diploid

YEST plate

G418 plate

DY47922 cross-1

Successful octad: 11

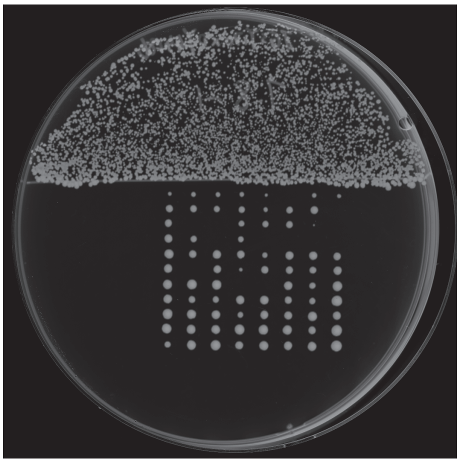

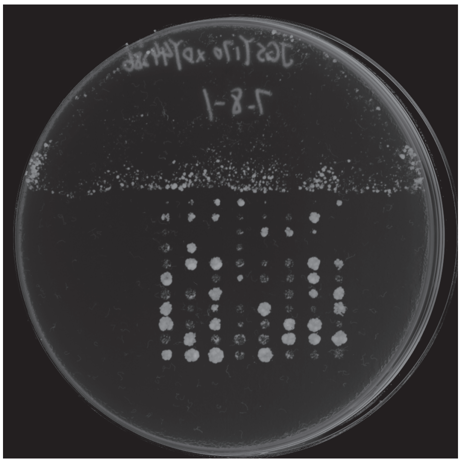

DY47922 cross-2

Successful octad: 11

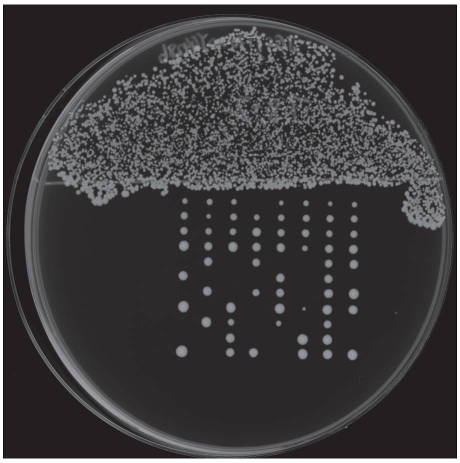

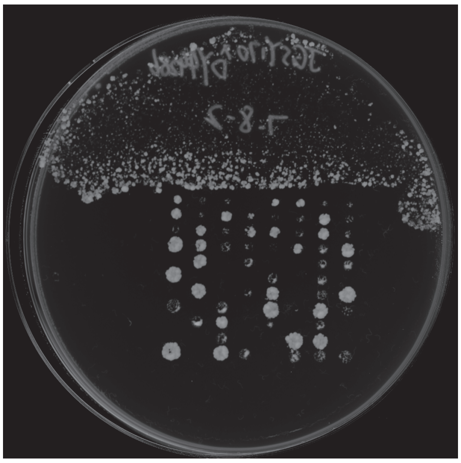

DY47922 cross-3

Successful octad: 11

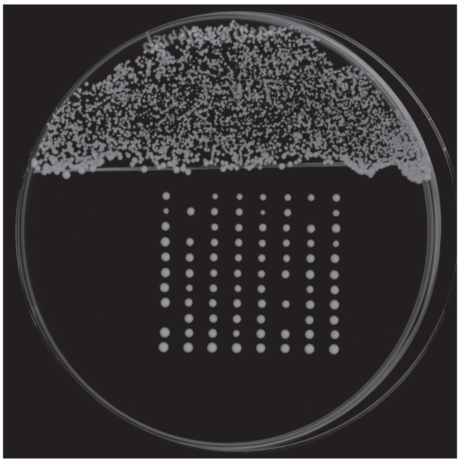

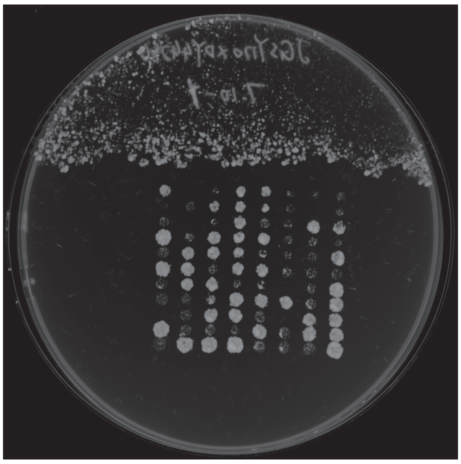

DY47922 cross-4

Successful octad: 11

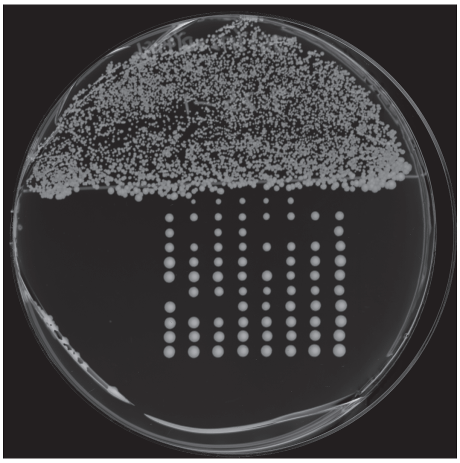

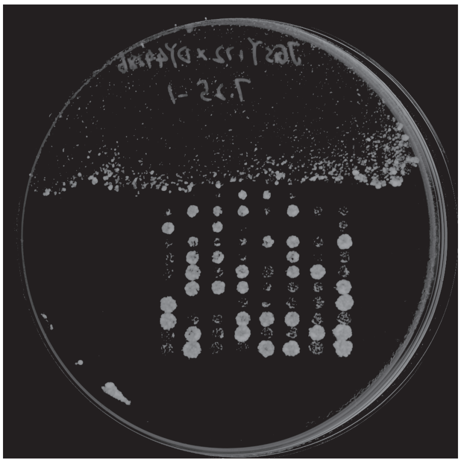

wtf60(SOCG_04742)Δ/wtf60(SOCG_04742)⁺ heterozygous diploid

YEST plate

G418 plate

DY47922 cross-5

Successful octad: 10

DY47922 cross-6

Successful octad: 11

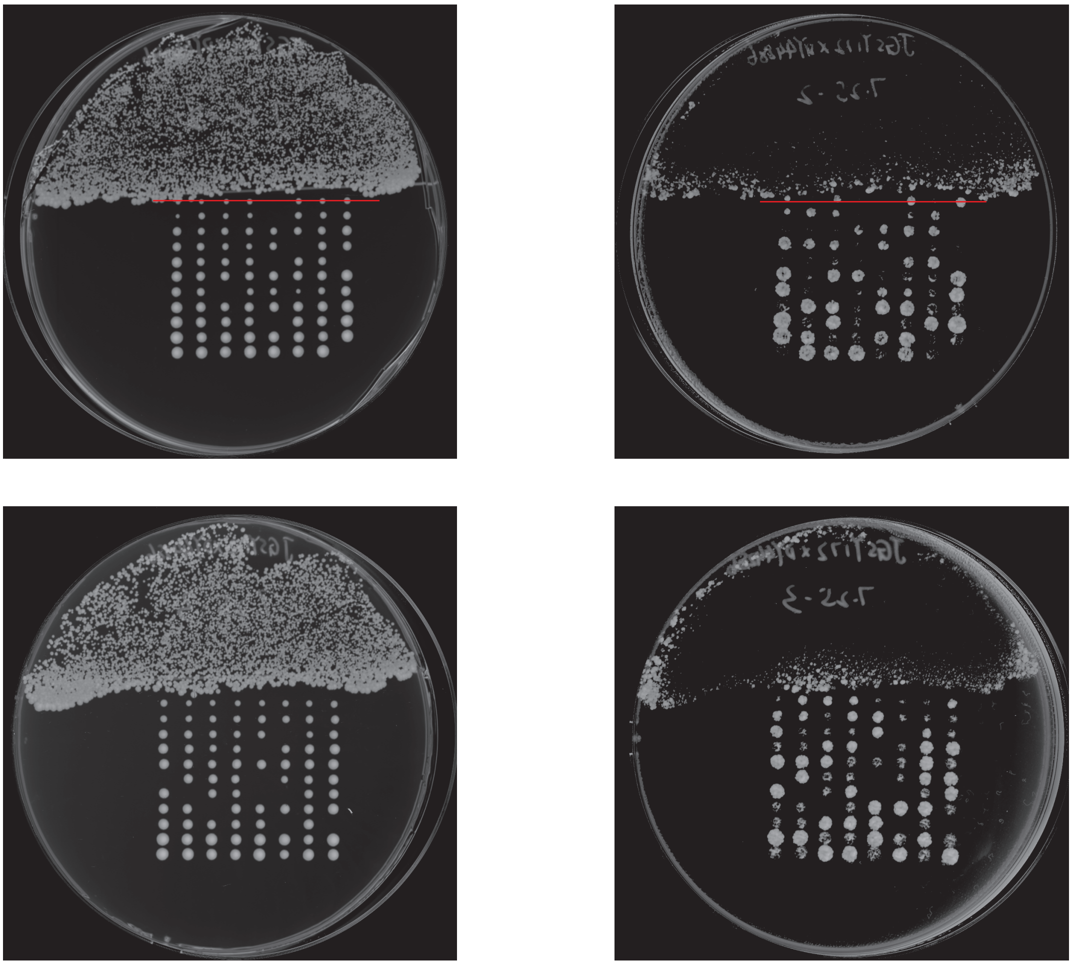

Supplement: Figure 9—figure supplement 3—source data 2. — wtf60+/wtf60Δ heterozygous diploid raw data files are shown as a pdf file with each cross in the upper left of the images. [file elife-81149-fig9-figsupp3-data2.pdf]
