# Supplementary material for: The wtf meiotic driver gene family has unexpectedly persisted for over 100 million years
Source: eLife. 2022 Oct 13;11:e81149. doi: 10.7554/eLife.81149 (PMC9562144; doi:10.7554/eLife.81149)

wtf60(SOCG_04742)Δ/wtf60(SOCG_04742)Δ homozygous diploid

	YEST plate	G418 plate
DY47921 × DY47922 -1<br>Successful octad: 11	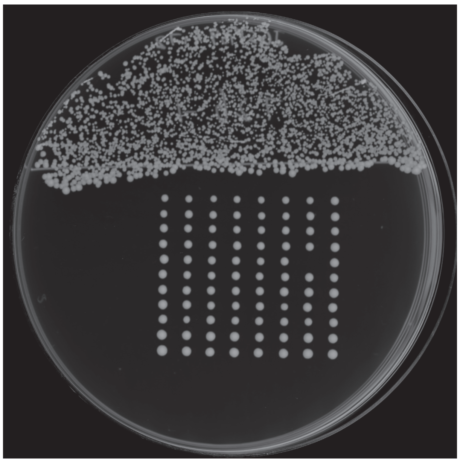	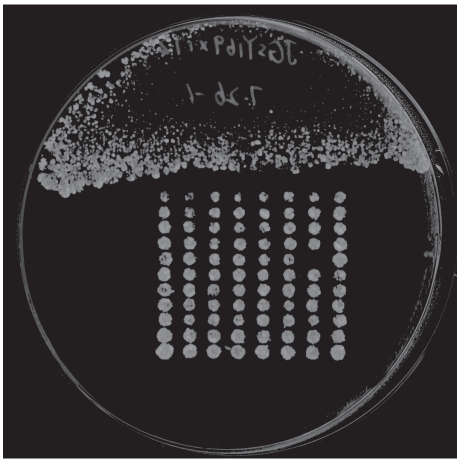
DY47921 × DY47922 -2<br>Successful octad: 11	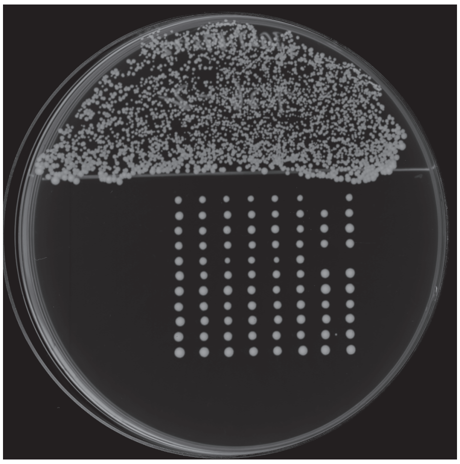	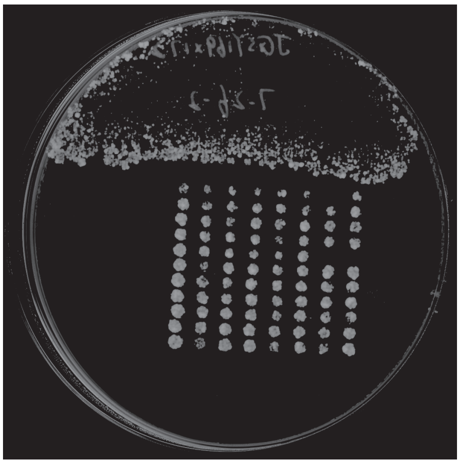
DY47921 × DY47922 -3<br>Successful octad: 10	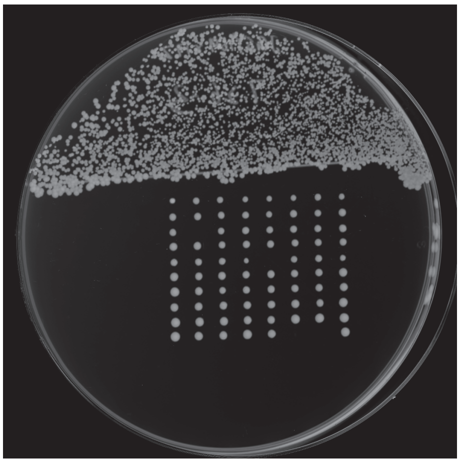	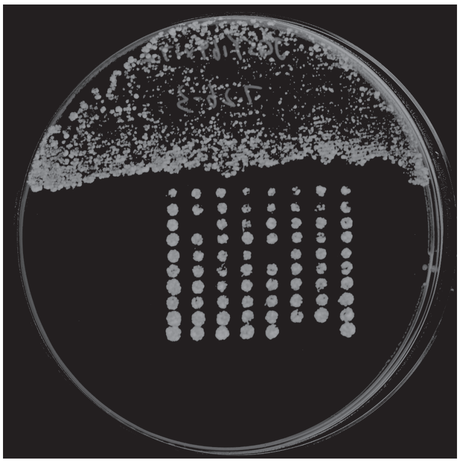
DY47921 × DY47922 -4<br>Successful octad: 11	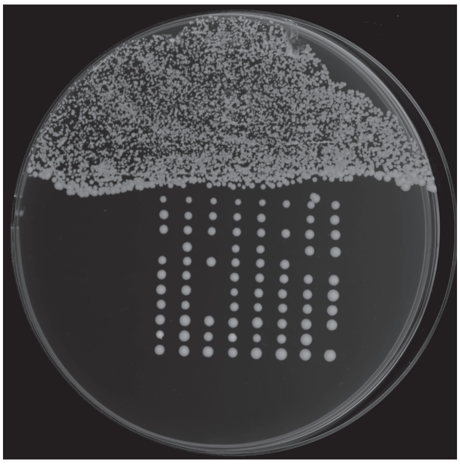	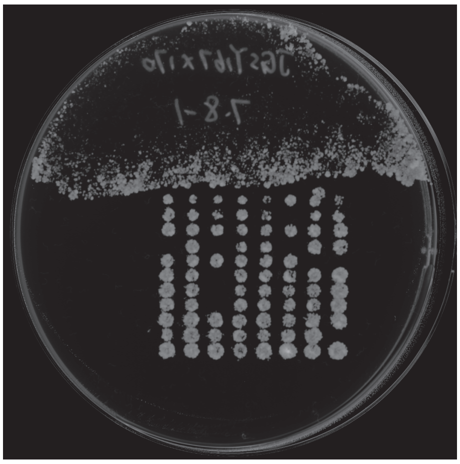

wtf60(SOCG_04742)Δ/wtf60(SOCG_04742)Δ homozygous diploid

YEST plate

G418 plate

DY47921 × DY47922 -5

Successful octad: 11

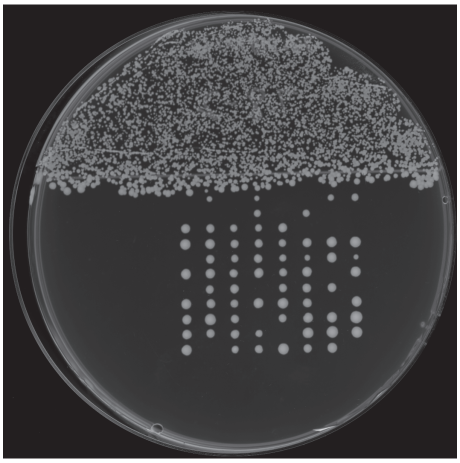

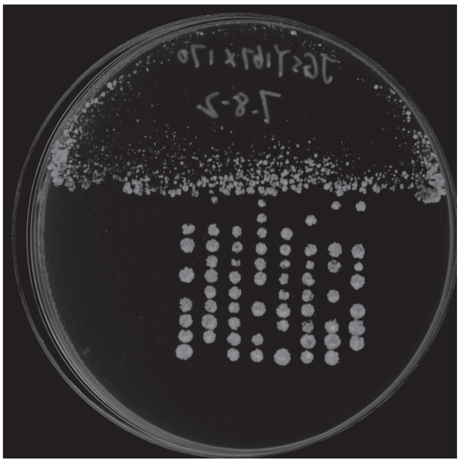

Supplement: Figure 9—figure supplement 3—source data 3. — wtf60Δ/ wtf60Δ homozygous diploid raw data files are shown as a pdf file with each cross in the upper left of the images. [file elife-81149-fig9-figsupp3-data3.pdf]
