# Supplementary material for: The wtf meiotic driver gene family has unexpectedly persisted for over 100 million years
Source: eLife. 2022 Oct 13;11:e81149. doi: 10.7554/eLife.81149 (PMC9562144; doi:10.7554/eLife.81149)

wtf46(SOCG_00084)Δ/wtf46(SOCG_00084)$^{+}$ heterotygous diploid

	YEST plate	G418 plate

DY47907 cross-1
Successful octad: 11

DY47907 cross-2
Successful octad: 8

DY47907 cross-3
Successful octad: 10

DY47907 cross-4
Successful octad: 10

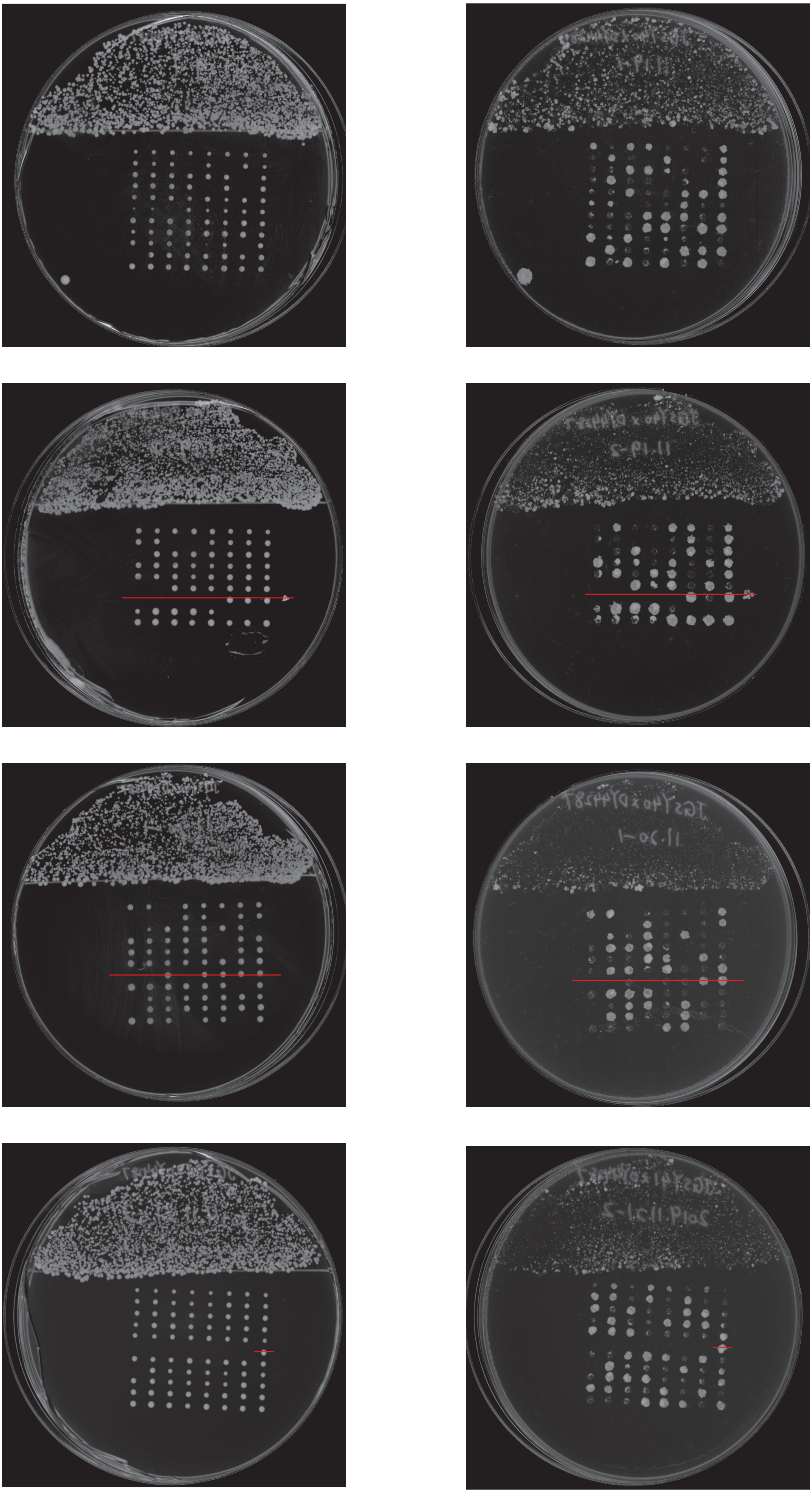

wtf46(SOCG_00084)Δ/wtf46(SOCG_00084)+ heterotygous diploid

YEST plate

G418 plate

DY47907 cross-5

Successful octad: 11

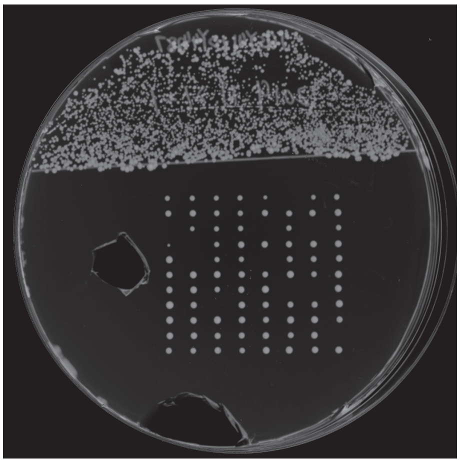

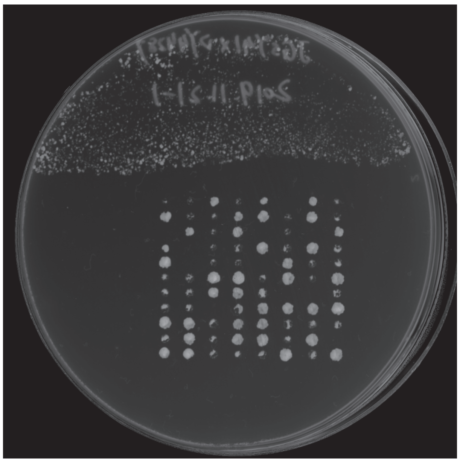

wtf46(SOCG_00084)Δ/wtf46(SOCG_00084)$^{+}$ heterotygous diploid

YEST plate

G418 plate

DY47908 cross-1

Successful octad: 8

DY47908 cross-2

Successful octad: 6

DY47908 cross-3

Successful octad: 10

DY47908 cross-4

Successful octad: 11

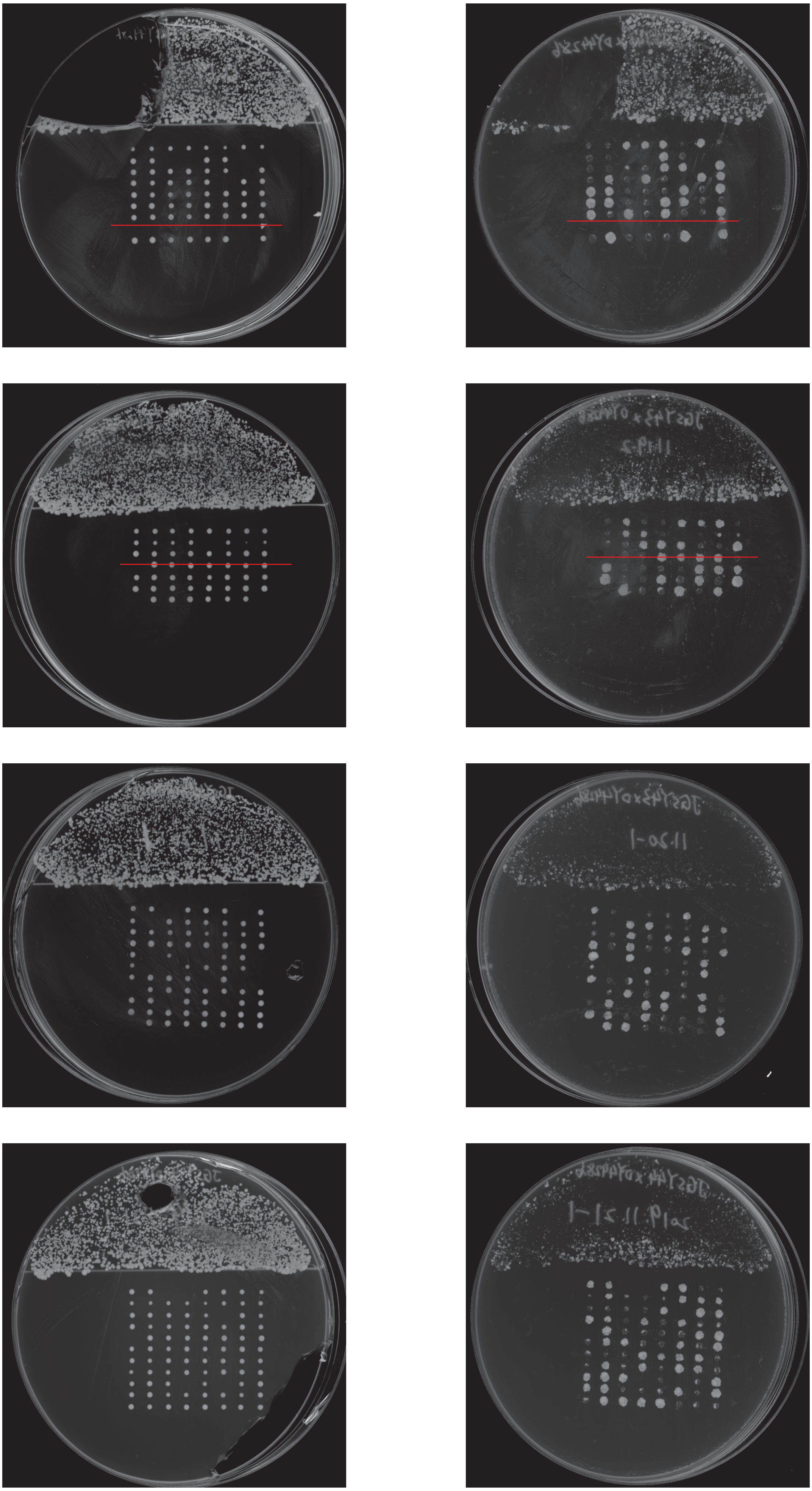

Supplement: Figure 9—figure supplement 4—source data 2. — wtf46+/wtf46Δ heterozygous diploid raw data files are shown as a pdf file with each cross in the upper left of the images. [file elife-81149-fig9-figsupp4-data2.pdf]
