# Supplementary material for: The wtf meiotic driver gene family has unexpectedly persisted for over 100 million years
Source: eLife. 2022 Oct 13;11:e81149. doi: 10.7554/eLife.81149 (PMC9562144; doi:10.7554/eLife.81149)

wtf46(SOCG_00084)Δ/wtf46(SOCG_00084)Δ homozygous diploid

YEST plate | G418 plate

DY47907 × DY47908 -1

Successful octad: 10

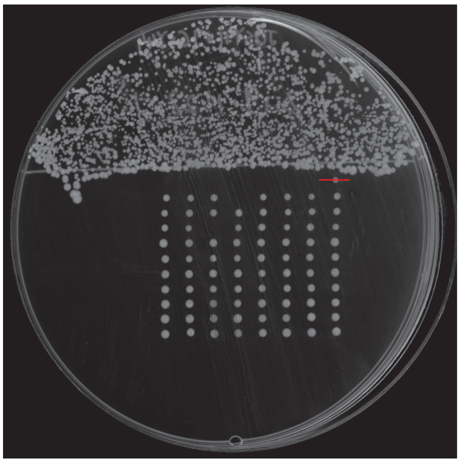

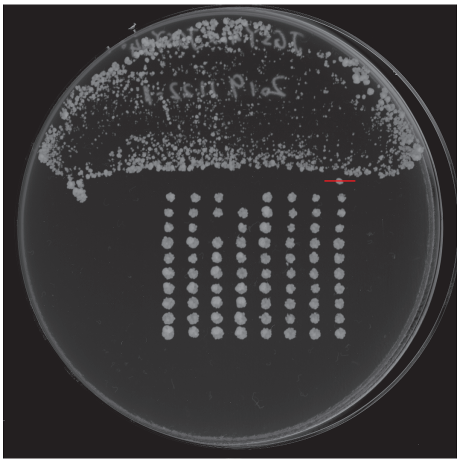

DY47907 × DY47908 -2

Successful octad: 11

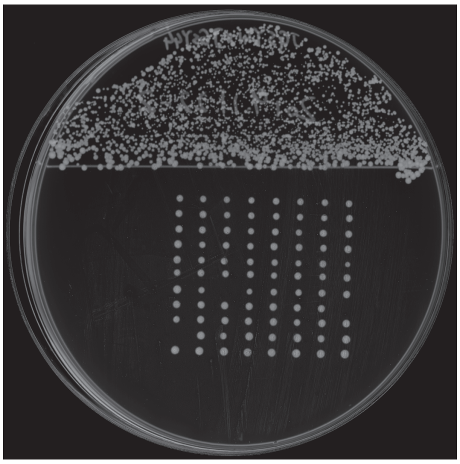

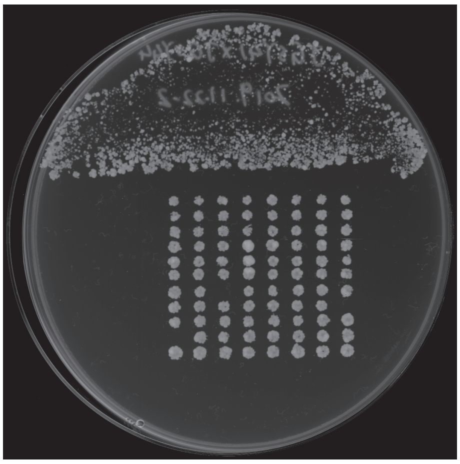

DY47907 × DY47908 -3

Successful octad: 10

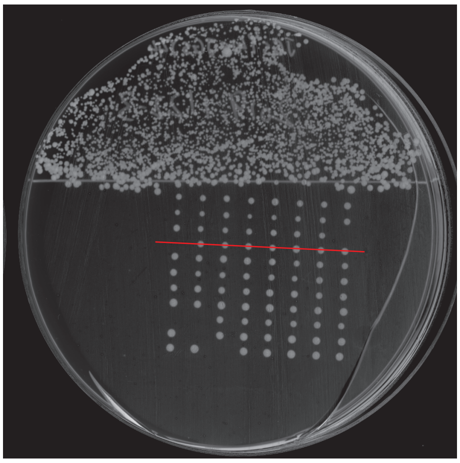

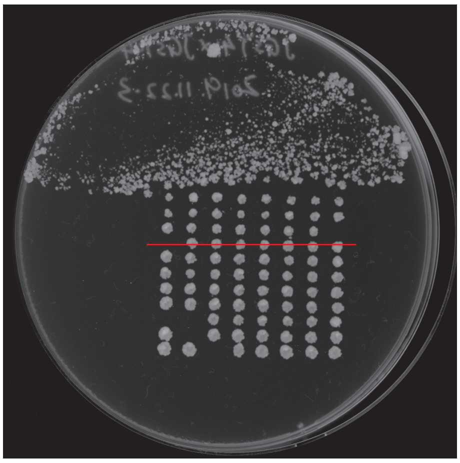

DY47907 × DY47908 -4

Successful octad: 5

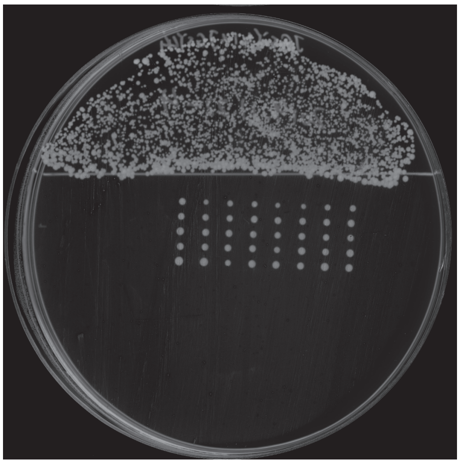

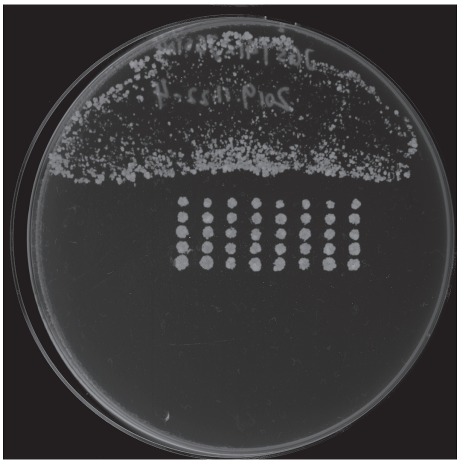

wtf46(SOCG_00084)Δ/wtf46(SOCG_00084)Δ homozygous diploid

YEST plate | G418 plate

DY47907 × DY47908 -1

Successful octad: 11

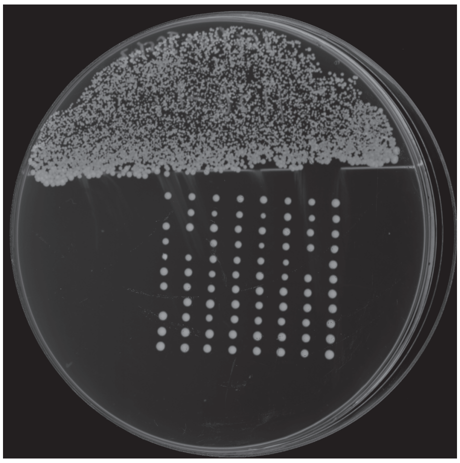

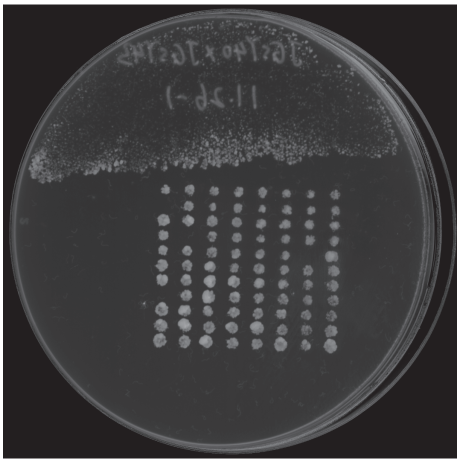

DY47907 × DY47908 -2

Successful octad: 11

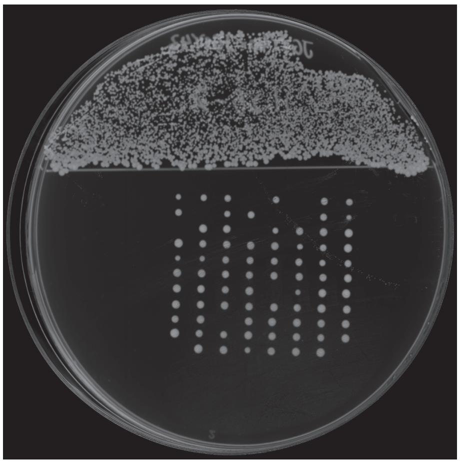

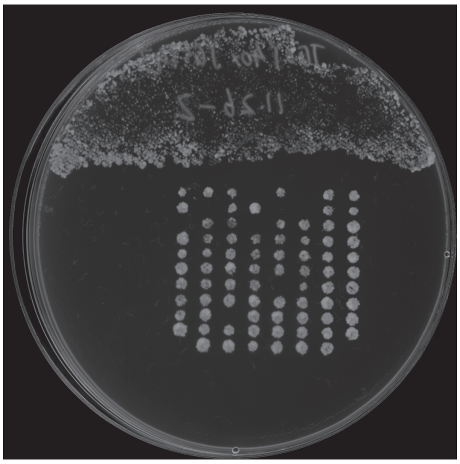

Supplement: Figure 9—figure supplement 4—source data 3. — wtf46Δ/wtf46Δ homozygous diploid raw data files are shown as a pdf file with each cross in the upper left of the images. [file elife-81149-fig9-figsupp4-data3.pdf]
