# Supplementary material for: The wtf meiotic driver gene family has unexpectedly persisted for over 100 million years
Source: eLife. 2022 Oct 13;11:e81149. doi: 10.7554/eLife.81149 (PMC9562144; doi:10.7554/eLife.81149)

wtf21(SOCG_02322)Δ/wtf21(SOCG_02322)$^{+}$ heterozygous diploid

YEST plate

G418 plate

DY47925 cross-1

Successful octad: 10

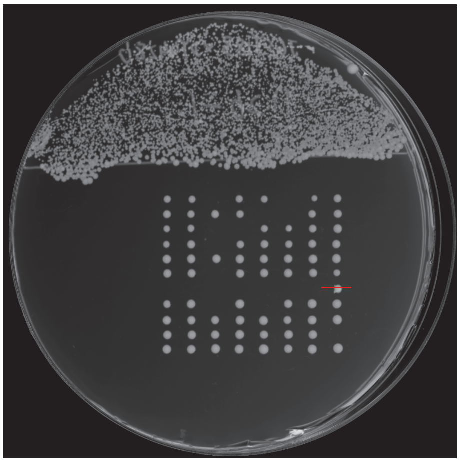

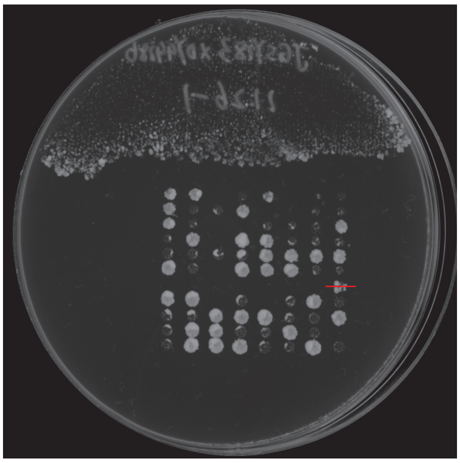

DY47925 cross-2

Successful octad: 10

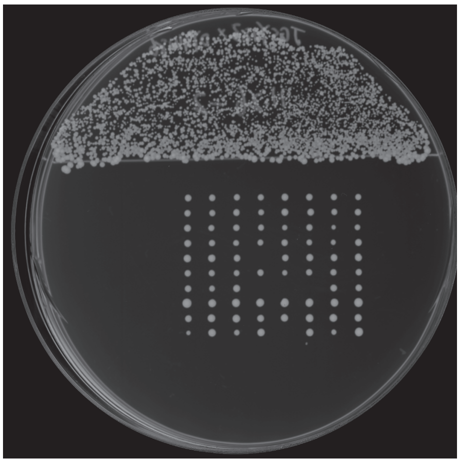

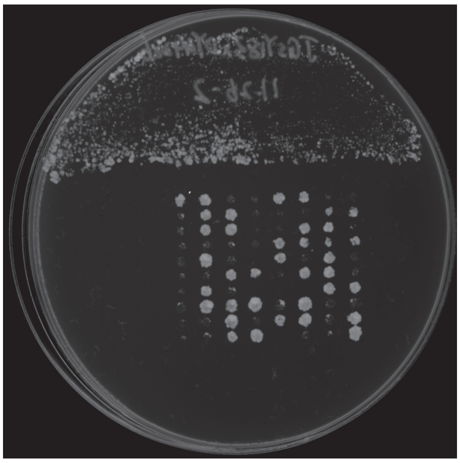

DY47925 cross-3

Successful octad: 10

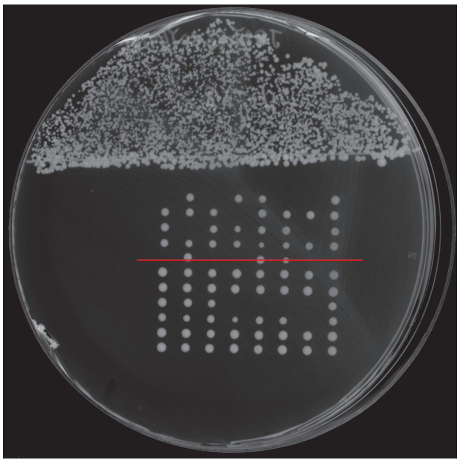

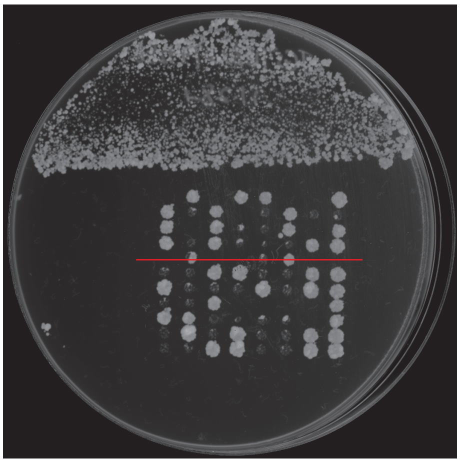

DY47925 cross-4

Successful octad: 11

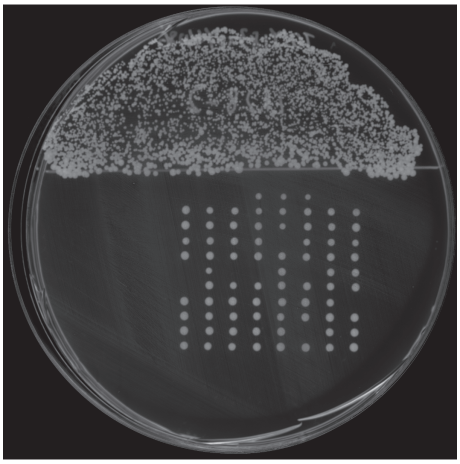

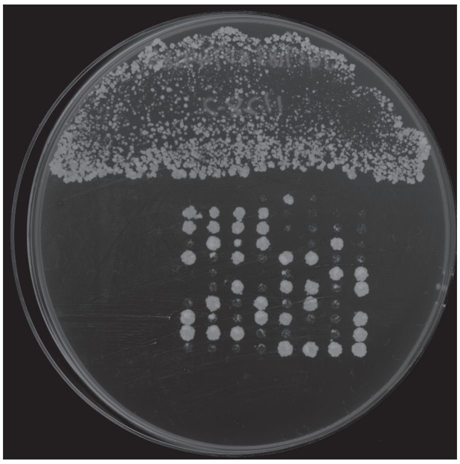

wtf21(SOCG_02322)Δ/wtf21(SOCG_02322)⁺ heterozygous diploid

YEST plate | G418 plate

DY47925 cross-5

Successful octad: 10

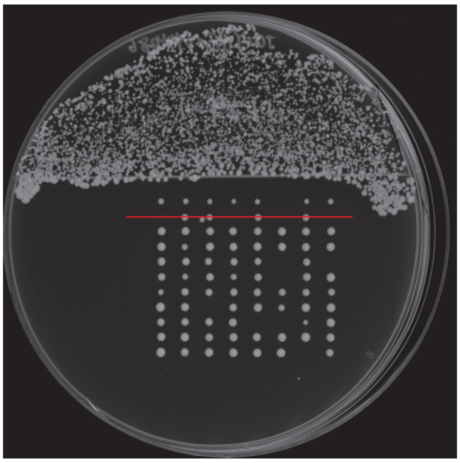

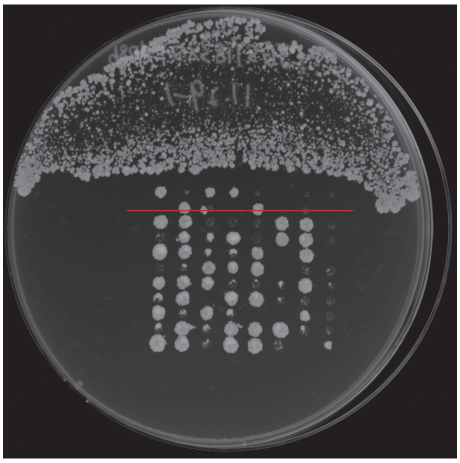

DY47925 cross-6

Successful octad: 10

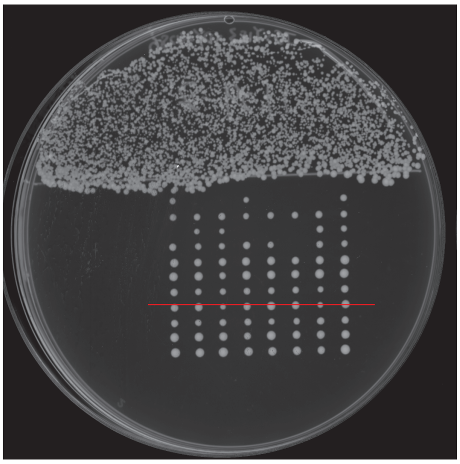

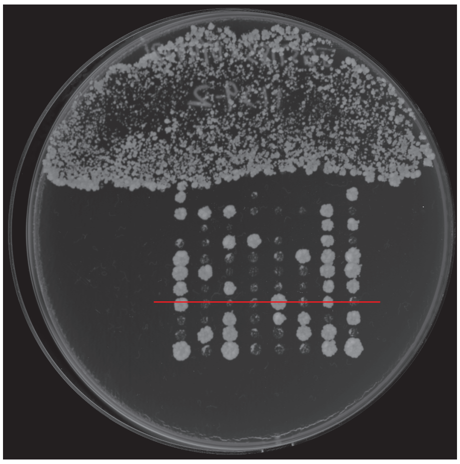

DY47925 cross-7

Successful octad: 10

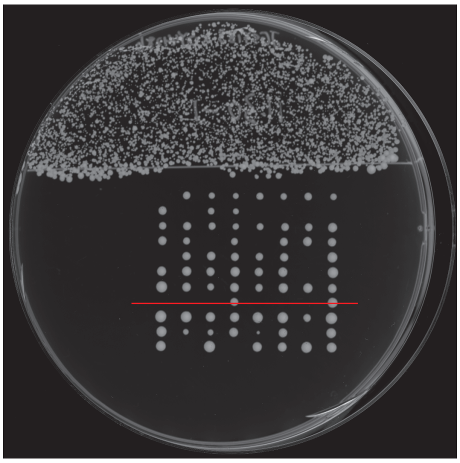

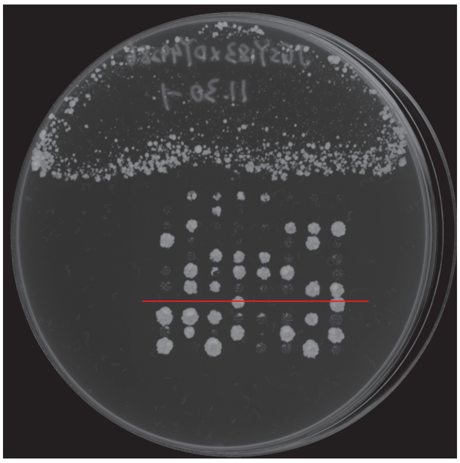

DY47925 cross-8

Successful octad: 9

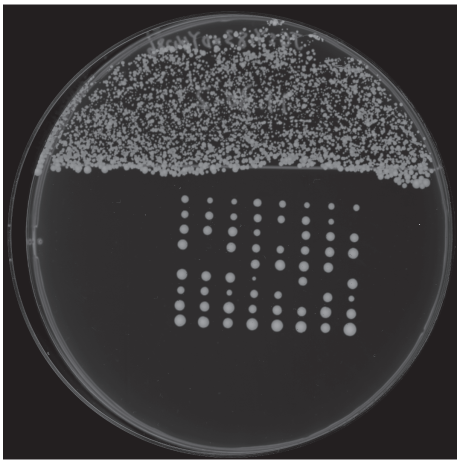

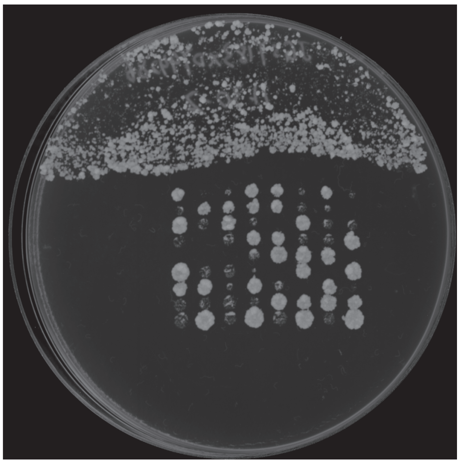

wtf21(SOCG_02322)Δ/wtf21(SOCG_02322)$^{+}$ heterozygous diploid

YEST plate | G418 plate

DY47926 cross-1
Successful octad: 11

DY47926 cross-2
Successful octad: 11

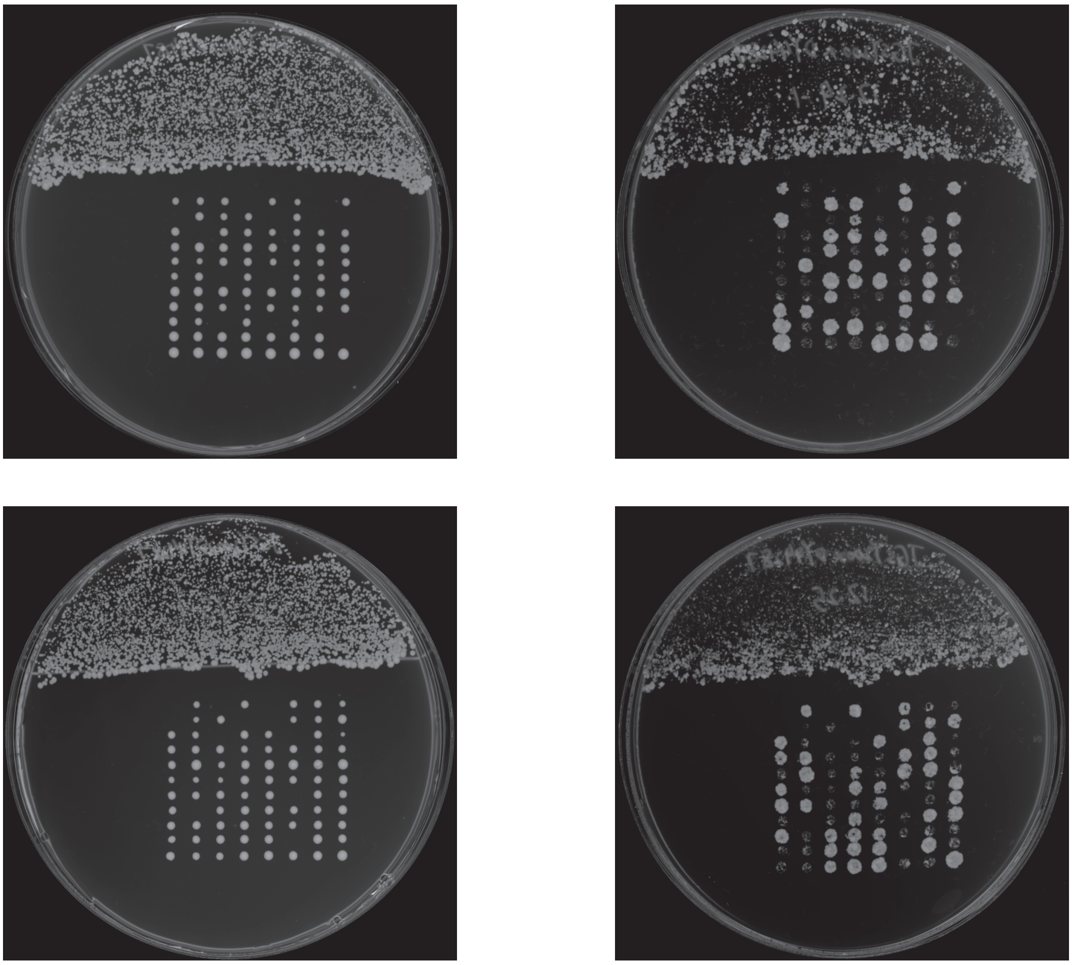

Supplement: Figure 9—figure supplement 5—source data 2. — wtf21+/wtf21Δ heterozygous diploid raw data files are shown as a pdf file with each cross in the upper left of the images. [file elife-81149-fig9-figsupp5-data2.pdf]
