# Supplementary material for: The wtf meiotic driver gene family has unexpectedly persisted for over 100 million years
Source: eLife. 2022 Oct 13;11:e81149. doi: 10.7554/eLife.81149 (PMC9562144; doi:10.7554/eLife.81149)

wtf21(SOCG_02322)Δ/wtf21(SOCG_02322)Δ homozygous diploid

YEST plate | G418 plate

DY47925 × DY47926 -1
Successful octad: 11

DY47925 × DY47926 -2
Successful octad: 11

DY47925 × DY47926 -3
Successful octad: 6

DY47925 × DY47926 -4
Successful octad: 10

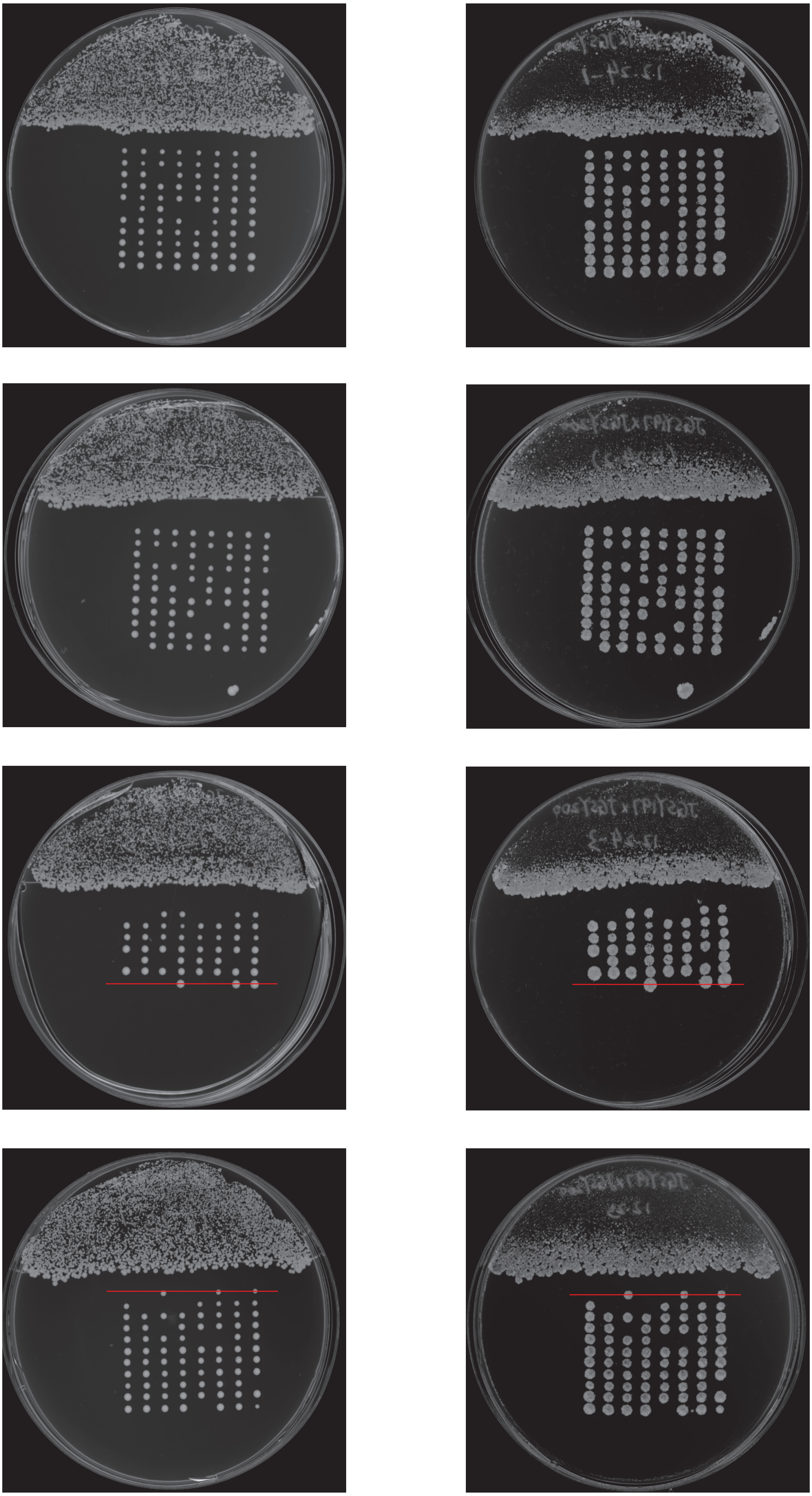

Supplement: Figure 9—figure supplement 5—source data 3. — wtf21Δ/wtf21Δ homozygous diploid raw data files are shown as a pdf file with each cross in the upper left of the images. [file elife-81149-fig9-figsupp5-data3.pdf]
