# Supplementary material for: The wtf meiotic driver gene family has unexpectedly persisted for over 100 million years
Source: eLife. 2022 Oct 13;11:e81149. doi: 10.7554/eLife.81149 (PMC9562144; doi:10.7554/eLife.81149)

wtf62(SOCG_04077)Δ/wtf62(SOCG_04077)+ heterozygous diploid

YEST plate | G418 plate

DY47923 cross-1

Successful octad: 9

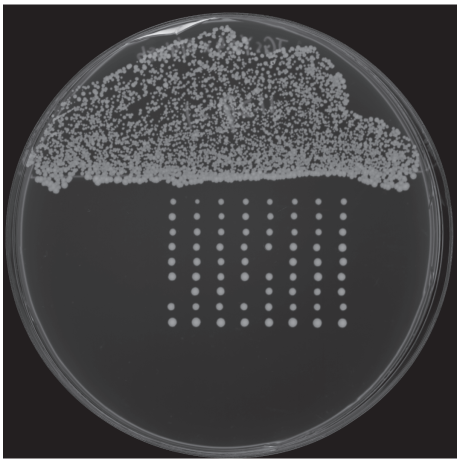

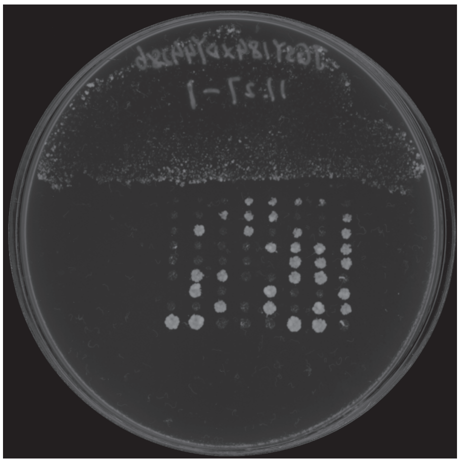

DY47923 cross-2

Successful octad: 11

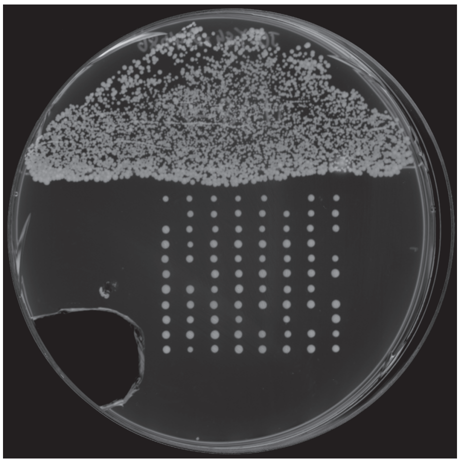

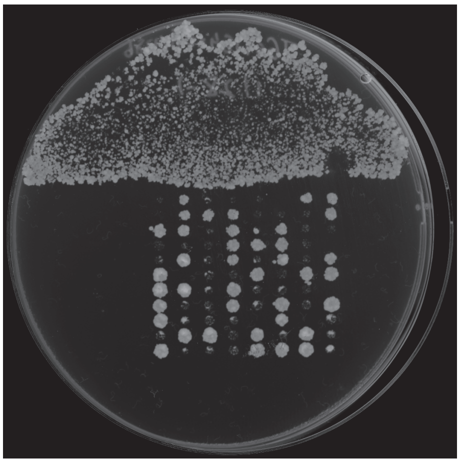

DY47923 cross-3

Successful octad: 11

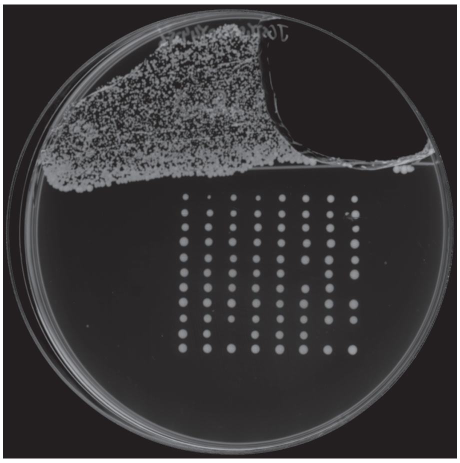

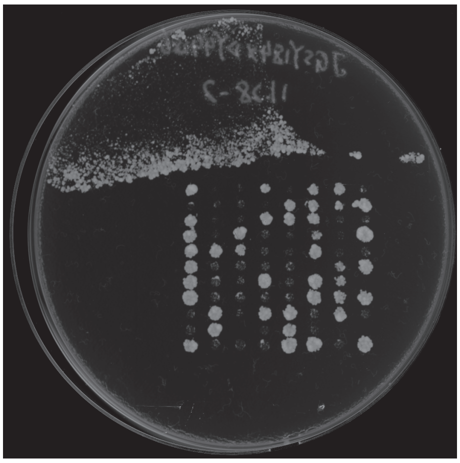

DY47923 cross-4

Successful octad: 5

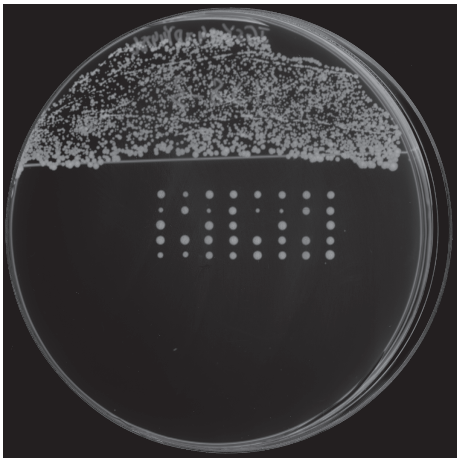

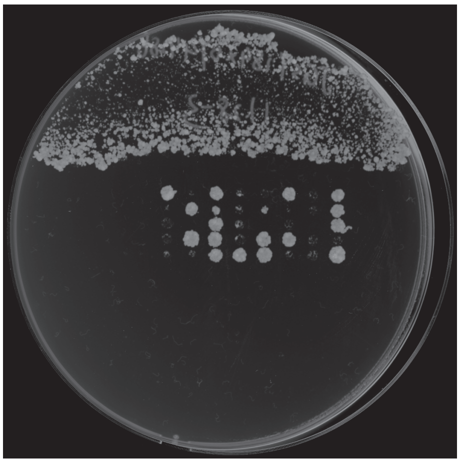

wtf62(SOCG_04077)Δ/wtf62(SOCG_04077)⁺ heterozygous diploid

YEST plate

G418 plate

DY47923 cross-5

Successful octad: 11

DY47923 cross-6

Successful octad: 10

DY47923 cross-7

Successful octad: 11

DY47923 cross-8

Successful octad: 10

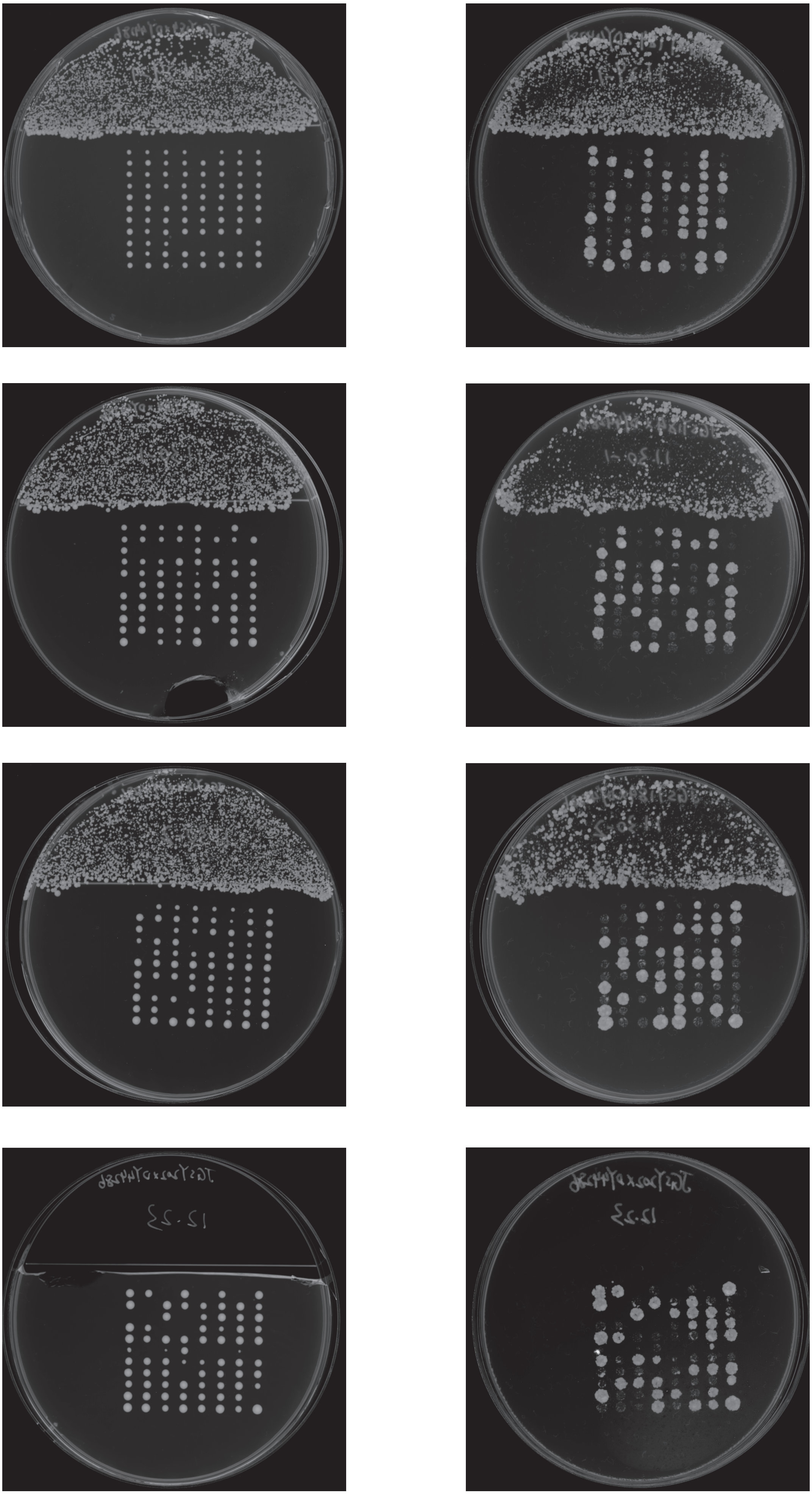

wtf62(SOCG_04077)Δ/wtf62(SOCG_04077)$^{+}$ heterozygous diploid

YEST plate

G418 plate

DY47924 cross-1

Successful octad: 11

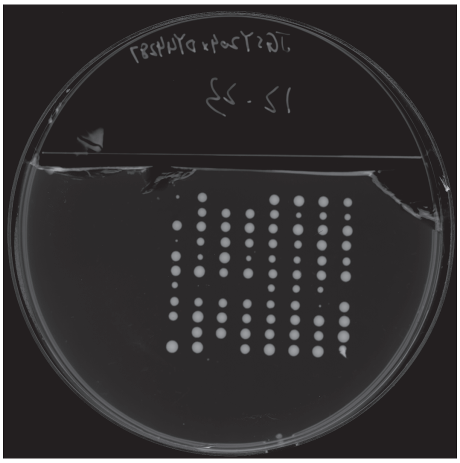

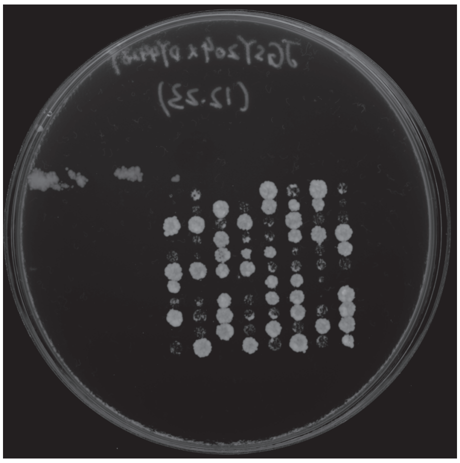

Supplement: Figure 9—figure supplement 6—source data 2. — wtf62+/wtf62Δ heterozygous diploid raw data files are shown as a pdf file with each cross in the upper left of the images. [file elife-81149-fig9-figsupp6-data2.pdf]
