# Supplementary material for: The wtf meiotic driver gene family has unexpectedly persisted for over 100 million years
Source: eLife. 2022 Oct 13;11:e81149. doi: 10.7554/eLife.81149 (PMC9562144; doi:10.7554/eLife.81149)

*wtf62(SOCG_04077)Δ/wtf62(SOCG_04077)Δ* homozygous diploid

| | YEST plate | G418 plate |
|---|---|---|
| DY47923 × DY47924 -1<br>Successful octad: 11 | 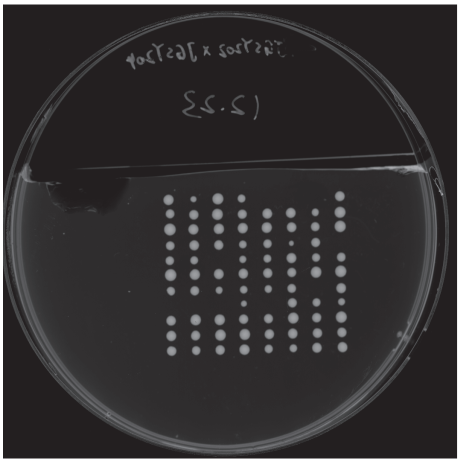 | 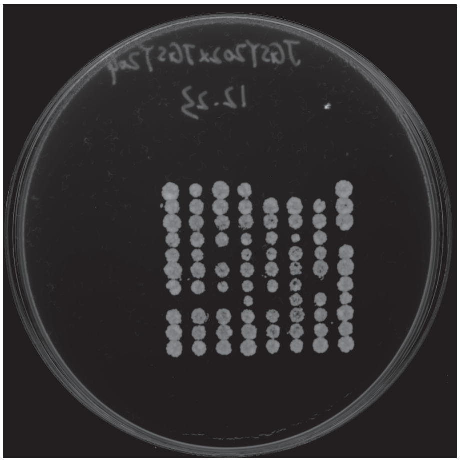 |
| DY47923 × DY47924 -2<br>Successful octad: 11 | 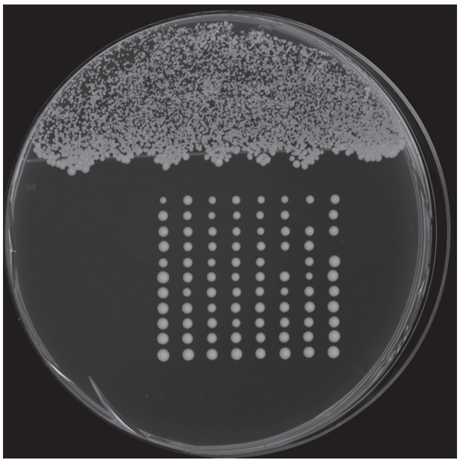 | 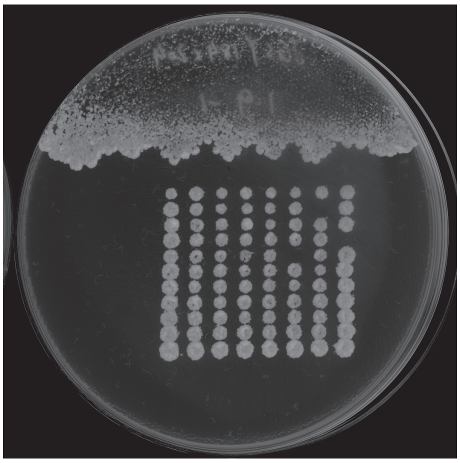 |
| DY47923 × DY47924 -3<br>Successful octad: 11 | 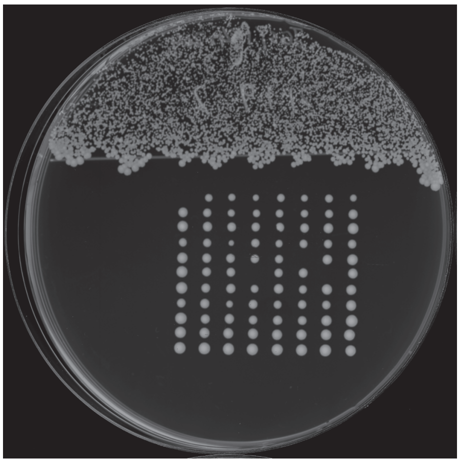 | 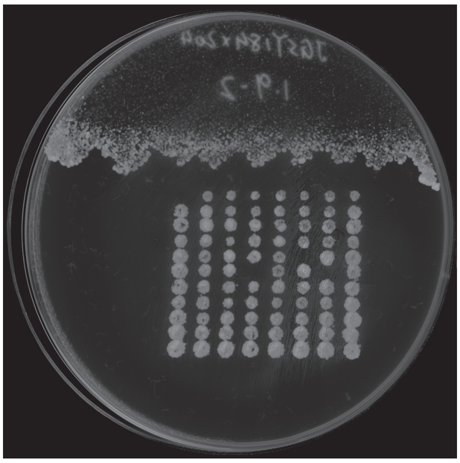 |
| DY47923 × DY47924 -4<br>Successful octad: 11 | 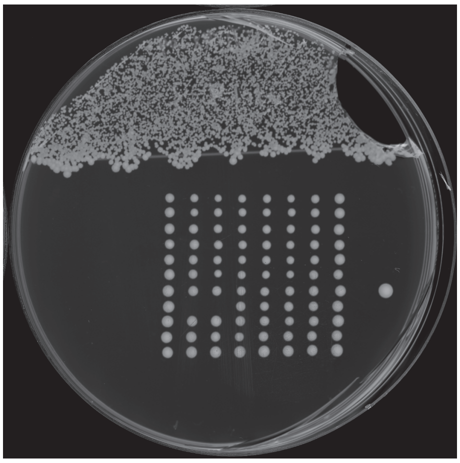 | 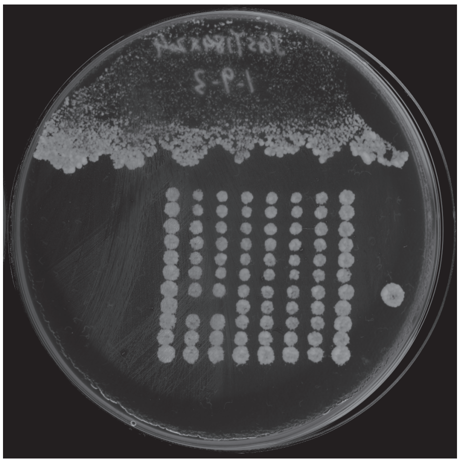 |

*wtf62(SOCG_04077)Δ/wtf62(SOCG_04077)Δ* homozygous diploid

DY47923 × DY47924 -5

Successful octad: 11

YEST plate

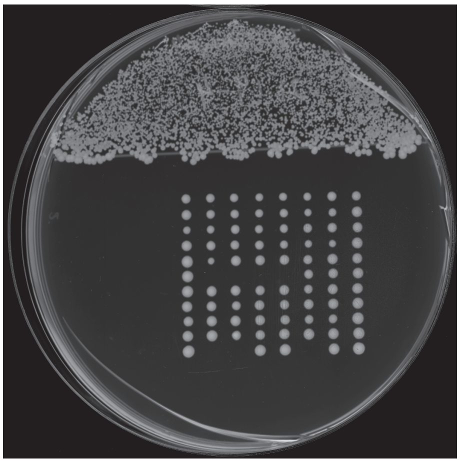

G418 plate

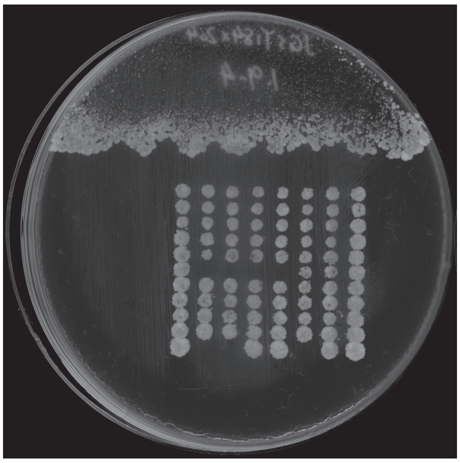

Supplement: Figure 9—figure supplement 6—source data 3. — wtf62Δ/wtf62Δ homozygous diploid raw data files are shown as a pdf file with each cross in the upper left of the images. [file elife-81149-fig9-figsupp6-data3.pdf]
